# Facial recognition lock technology for social care settings: A qualitative evaluation of implementation of facial recognition locks at two residential care sites

**DOI:** 10.3389/fdgth.2023.1066327

**Published:** 2023-03-03

**Authors:** H. L. Bradwell, K. J. Edwards, R. Baines, T. Page, A. Chatterjee, R. B. Jones

**Affiliations:** Centre for Health Technology, University of Plymouth, Devon, United Kingdom

**Keywords:** facial recognition, security, access, social care, safety, innovation, facial recognition technology, care home

## Abstract

**Background:**

There is limited literature on security and access for social care settings despite policy highlighting importance, and no published research exploring facial recognition lock technology (FRLT) for potential improvements. This study explored FRLT device implementation, use, barriers and benefits.

**Methods:**

One residential care home with 43 older adults and 68 staff members (Site A), and one supported living facility caring for six individuals with mental health issues with 18 staff members (Site B) were provided with FRLT for six months. Nine pre-implementation staff interviews explored existing access and security perceptions. Ten post-implementation staff interviews and one staff focus group were conducted; all were analysed using content analysis to understand, alongside process mapping, the use and impact of the FRLT. Interview participants included site care staff and other visiting healthcare professionals. We additionally report feedback from the technology developers to demonstrate impact of industry-academia collaboration.

**Results:**

Pre-implementation interviews highlighted issues with current pin-pad or lock-box systems, including; code sharing; code visibility, ineffective code changes, security issues following high staff turnover, lack of efficiency for visitors including NHS staff and lack of infection control suggesting requirement for innovation and improvement. Pre-implementation interviews showed openness and interest in FRLT, although initial queries were raised around cost effectiveness and staff skills. Following implementation, good levels of adoption were achieved with 72% and 100% (49/68 and 18/18) of staff members uploading their face at the two sites, and 100% of residents at Site B using the system (6/6). Additionally, Site B made a positive procurement decision and continues to discuss wider rollout. Post implementation interviews suggested FRLT was useful and acceptable for improving security and access. Benefits identified included staff/visitor time saving, enhanced security, team ease of access, resident autonomy and fewer shared touch points. Integration was suggested including with fire alarm systems, staff clocking in/out, and Covid monitoring to improve usefulness. The developers have since responded to feedback with design iterations.

**Conclusion:**

We identified concerns on security and access in social care settings, which warrant further exploration and research. FRLT could increase resident autonomy and reduce staff burden, particularly considering frequent multi-agency health and care visits.

## Background

### The care sector

Worldwide, the population is aging (1), increasing the number of people requiring complex care due to high levels of dependence, dementia and comorbidity (2–4) and consequent need for social care services (4). Social care staffing is also facing a crisis. For example, in the United Kingdom (UK) the adult social care sector pre-pandemic and pre-Brexit had 111,000 (8%) vacancies and a turnover rate of 31% (4, 5). Staff shortages and turnover have worsened since, with vacancies reportedly at 10%, contributing towards the “*greatest workforce crisis in history”* (6, 7).

While there has been increasing interest in the use of technologies to aid with the aging population and delivery of services (1), there has been limited focus on its use to enhance security and site access. Security, although complex, can be defined as provision of a predictable and stable environment, where a person or group of people can pursue their daily activities without disruption or harm, or fear of disturbance or injury (8, 9). As discussed by Ashurst (10), it is vital for care home residents and staff to feel they themselves and their belongings are safe and secure. As residential care settings house a large number of often vulnerable individuals, sites are designed to be secure (10), with exits alarmed, security cameras often in place and a reception often only accessible using pre-programmed entry code on pin-pad or permission granted by staff (10, 11). Access is also a complex concept, empirically meaning who gets use or benefit from what, in what ways and when (12). In the current context, access refers to physical entry to the secure care site for the purpose of visiting, work or health care provision. Visitors or businesses requiring access to care homes are often asked for proof of identity, presenting ID cards for inspection, signing fire registers and visitors' books on arrival and departure (10). Such requirements are often cumbersome and time consuming. The accuracy of such records is also essential in the case of any incidents (10), but our own time researching in care homes and discussing with stakeholders identified a general lack of diligence from visitors.

Darton (13) found all relatives suggested security was one reason for their choice of home, along with care for health needs, staff friendliness, homeliness, standard of care and cleanliness. Although less than half of residents stated security as a deciding factor, 88% of them expected the home to be secure and 87% expected to feel safe. Security in some care homes involves a careful balance between autonomy for residents and safety (11). The Care Quality Commission (CQC) also highlights a safety first approach (14). This risk management ethos has led to reliance on pin-pads and other forms of security, which may be linked to low levels of resident wellbeing, due to subsequent limitations in access to outdoor space or pleasurable activity (11, 15). Pin-pads and other high-touch shared surfaces may also be a source of infection transmission.

Evans noted in 2018 that little research had been conducted to gather views of stakeholders on physical access and perceptions of security for care homes (11). We have found little to change that since, despite the advances in technology.

### Facial recognition technology for care sector security

While technology in care homes to support care provision is receiving increasing research interest (16), and there is valuable research available on care home design (17), structure and management (11), there is to date no research exploring the potential for technological security solutions, and specifically facial recognition technology (FRT).

FRT has become a well-accepted biometric method, due to the particular uniqueness and distinguishability of faces (18). The technology has thus been studied extensively for use in security, surveillance and identification (18). The use of facial recognition lock technology (FRLT) for enhanced security is becoming established for other sectors, for example, in schools, factories, airports, shopping areas and government buildings (19). Beyond use for security, more recently attention has also focused on the potential for FRT as a touchless method of entry to enhance infection control safety in hospital during the pandemic (20), although no implementation has been reported. Although the technology proposed is similar to that discussed here, the hospital context differs somewhat from the social care settings trialling FRLT in our study. Hospitals receive a higher volume of visitors than care homes or other residential care settings, where generally the same residents, staff and family members require daily access, compared with high turnover of patients within a hospital and larger staff workforces. In other FRLT work, previous research by Sander and Oo (21) and Yedulapuram et al. (22) report on the design of facial recognition lock systems from a technical perspective, but lack any data on implementation, use and end-user perceptions. We aim to address this with novel insight into an evaluation of real-world implementation of FRLT into care settings.

FRT is not without limitations however, with one concern relating to privacy. Some literature reports on the use of thermal FRT, to identify individuals with a fever and limit contagion of Covid-19, warning about the privacy implications and need for policy to regulate use (23). When considering FRT in any setting, there must be adequate consideration given to possible harms, such as use by governments to repress opposition, identify protestors and disrupt public freedom (24). This potential for harm may explain some of the negative public perceptions of FRT. In a survey of 1000 individuals, the London Policing Ethics Panel (25) reported that over a third were concerned about their privacy in relation to live FRT. Young people demonstrated most discomfort; 38% of 16–25 year olds would avoid events using live FRT (25). There are additionally documented challenges in recognition of aging faces, and a stream of work underway exploring solutions to improve accuracy (26). However, there is limited literature available on perceptions of employing FRT within occupational settings, specifically, health and care.

Considering the combination of challenges facing the social care sector worldwide, the uptake in technology to support service provision, the lack of research into social care security and innovations to this regard, and the controversial perceptions on FRT, research into stakeholder perceptions and a FRLT implementation evaluation will contribute towards current understanding, practice and policy.

## Method

### Design

We used a qualitative, exploratory design to understand pre-implementation perceptions towards security and access in social care among key stakeholders, then to provide a FRLT implementation evaluation at two sites over six months, from February 2021.

### Ethics

This study received ethical approval from the Faculty of Health Ethics Committee at the University of Plymouth. Participants provided written informed consent after receiving written (participant information sheet) and verbal information on the researcher's aims and the study purpose.

### Setting

Site A was a residential care home caring for 43 older adults, those with dementia and mental health needs. It had 68 staff members and is part of a larger chain providing residential care at several sites regionally.

Site B was a supported living facility caring for six individuals with mental health issues with 18 staff members. Site B is part of a larger chain providing housing and support for people with disabilities and mental health conditions through several sites nationally.

Both sites are situated in Cornwall, UK.

### Procedure

Both sites received a facial recognition camera and associated system to control the main entrance lock and staff, residents (those with autonomy to leave the facility) and visitors were invited to upload their face image to use the system by sending a “selfie” to the site staff. Initially, staff took their own “selfies” and sent them to the TouchByte developers for upload. Later, key staff within the site were granted approval rights and capacity to upload photos into the web-based account. Residents with capacity were supported by staff to take a photo and upload their picture. Residents without capacity and autonomy to leave the sites unassisted were not uploaded onto the system. The facial recognition entry system was available alongside usual security systems (pin-pads), to ensure open choice and back-up in case of system failure. Both sites trialled the FRLT for six months before providing feedback (August/September 2021).

### Data collection

To collect an understanding of perceptions towards current access and security within the care sector, we conducted pre-implementation interviews (*via* Zoom or face-to-face depending on participant preference), during January and February 2021. During these interviews we also explored perceptions of FRLT.

Following the implementation period, we conducted post-implementation interviews to establish user-experience of FRLT *via* interviews and focus groups in August/September 2021. While the majority of data was collected in individual interviews (*via* Zoom or face-to-face on site), four staff at Site B provided post-implementation feedback *via* face-to-face focus group at Site B rather than individually, due to feasibility (as the site is a busy setting, staff felt it was more suitable to join together for a break and conduct their focus group then rather than take staff away at different points throughout the day).

Pre and post interviews and focus groups were conducted by two researchers experienced in qualitative data collection (HB, KE), with one researcher leading discussions and the other raising useful additional questions where relevant. No one was present other than researchers and participants. The interview/focus group questions followed semi-structured prompts available in Supplementary Appendix A1.

We additionally conducted process mapping to compare access to care sector sites pre/post implementation *via* interviews at the end of the pre-post interviews detailed above. (pre-implementation process mapping January/February 2021, post-implementation process mapping August/September 2021).

Process mapping is a form of interviewing and visualisation used to create understanding of current processes and pathways of working. The process is used within the NHS and helps identify and expose issues or inefficiencies in within specific processes and is most often used before making service changes (27). Therefore, this method suited the aims of the current study, which required understanding of a specific process before and after a change to FRLT, further to exposure of any inefficiencies. Process mapping is typically conducted in person with “sticky notes” and marker pens. The sessions begin with a high-level map of the start point and end point (e.g., start point of arriving at a care home and end point of continuing with the desired task within the home). Participants then provide steps required to get from start to end point, and any additional details such as time required for each step or barriers encountered.

For this study, process mapping was conducted directly after pre/post interviews as a visual, interactive activity over the Zoom platform using the Miro interface (The Miro.com website allows multiple users to simultaneously and remotely interact with a live visual document, where you can add text, “sticky notes” and shapes). Participants were asked in these interviews to provide step-by-step accounts (prompts available Supplementary Appendix AS2) of access to their relevant care site, while the researcher produced “sticky notes” to document the process. Participants could view the live Miro board, add steps, remove steps and add comments and notes on time taken for each step. The researchers consolidated all “sticky notes” to demonstrate the general process in the maps presented. The process maps, once consolidated, were shown to four further participants during the post-implementation focus group) to check validity of our interpretation and ensure they accurately reflected the steps encountered to gain access to settings.

Finally, data on the number of active users was collected to indicate rates of adoption (number of staff using the system) *via* log data from technology developers (August/September 2021) and procurement decisions by the implementation sites was collected *via* follow-up conversation (November 2021).

### Materials

**Figure 1:**
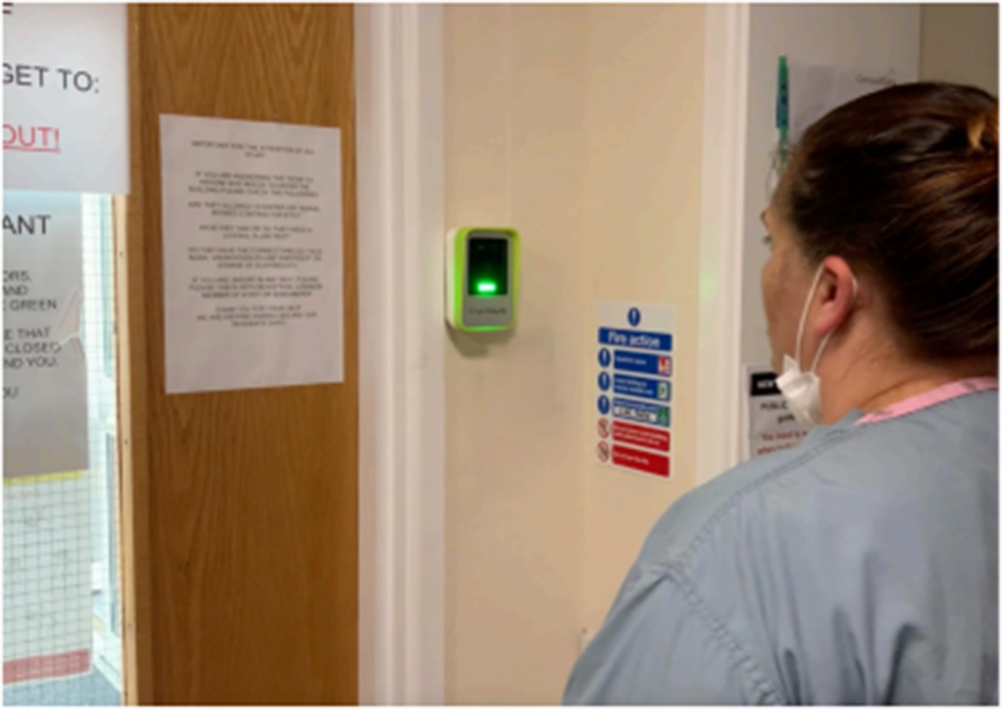
Touchbyte FRLT wall mounted camera displaying green indicator light.

The FRLT comprised a camera inside a wall-mounted device, positioned at the main entry door (Figure 1). The height positioning of the camera was selected to ensure accessibility based on 5th percentile female to 95th percentile male. The camera has a wide vertical view (approximately 125 degrees). The positioning therefore aims to capture a range of physical statures from a wheelchair user or 8–11 year old 5th percentile female to a standing 95th percentile male (plus 25 mm allowance for shoes) and above. The device and system were developed by a UK company, TouchByte (28). The device displays status indicator lights, which flash white when “checking” a face, red for no entry and green for entry approved. Faces can be uploaded to the system *via* a selfie taken on the individual's smartphone and sent *via* the internet to be uploaded, with the system checking it for suitability and informing the individual. Two staff members on each site (manager and administrator) within the organisation have approval rights for uploaded faces. Residents who uploaded to the system were supported by staff to take an appropriate photo and send it for upload. Access rights can be set for specific times (eg. a doctor can access during pre-defined appointment times only, or family can access only at allocated visiting times), and access can be revoked at any point (eg. for staff leaving). The system also includes some safety critical features, such as immediate access for paramedics and other emergency services *via* a barcode to display to the camera to avoid any delays in the case of an emergency. Although cost of the system can vary greatly based on a number of site-specific factors, including volume of users, features required, number of doors, to provide an indication of cost, an implementation similar to that at Site B would cost in the region of £950 + VAT for the hardware and a monthly fee of around £45 for software subscription and ongoing support.

### Data analysis

Audio recordings from interviews and focus groups were transcribed verbatim and analysed using content analysis on NVivo 12 software, involving systematic coding and categorising of text (29). Four researchers (HB, KE, RB, TP – all experienced qualitative researchers *via* their PhDs and currently digital health researchers), undertook the qualitative analysis, and as prescribed by Elo and Kyngäs (29), undertook a process of data immersion to familiarise with the entire data set, producing initial codes within the data, grouping codes, generating categories, and reporting, with a focus on manifest content. Researchers initially produced codes individually, and then engaged in a series of meetings to discuss codes, ensure consensus on representing the data set accurately, and agreeing on code groups and categories. The use of consensus among four qualitative researchers reduces some subjectivity and supports greater validity of interpretation. Quantitative data on the number of active users at each site were obtained from the technology developers. Process maps were created collaboratively with participants, and the final maps were produced by two researchers (HB, KE) summarising all process steps into a coherent map. The final process maps are displayed visually.

### Participants

In total, we collected qualitative data from 14 participants (Table 1), before and after implementation of FRLT to access two residential social care sites. Participants were recruited *via* opportunistic sampling, whereby potential participants were those involved in visiting or working at the implementation sites (in any capacity including as staff, carers, visitors, health professionals or managers). No family members took part due to visiting restrictions in relation to Covid-19 at the time of this study. Participants were approached by researchers by email and follow-up Zoom call (with contact details provided by staff on site). The sample size was dictated primarily by the number of staff and visitors to these sites willing to participate. The implementation sites were chosen as local care facilities, recommended for approach by the researchers by our network of health and care stakeholders. The researchers have all worked with social care settings for a number of years, so have good understanding of the general workings but had no prior relationships with the study sites. Data collection with the participants included 14 individual interviews, one focus group with four participants and five process mapping sessions following directly after interviews (validated in person in the focus group by a further four participants) (Table 1). Data collection sessions typically lasted 60–90 min.

**Table 1:** Stakeholder participants for data collection.

ID	Org	M/F	Role	Site	Data Collection		
					Pre	Post	Process
1	A	F	Senior management team member – regular visits to several care homes, manages all new and existing residents and transitions	A	X	X	X
2	A	M	Learning, development and compliance staff member - several visits to several care homes each month	A	X	X	
3	A	M	Non-executive director of care service – oversight and insight on residential and domiciliary care services	A	X		
4	A	M	Senior staff member in care catering team – regular visits to one care home, as required visits to several care homes	A	X	X	X
5	B	M	Managing director – oversight and management of domiciliary care service	A	X		
6	C	F	NHS district nurse – regular visits to multiple care homes and domiciliary care customers	Both	X		
7	A	F	Fundraising Manager – regular visits to multiple care homes, funding insight for residential and domiciliary care	A	X		
8	D	F	Social care academic – regular visits to multiple care homes	Both	X	X	X
9	A	F	Dementia team staff member – regular visits to multiple care homes	A	X	X	X
10	E	F	Team leader carer – daily visits to the supported living home	B		X	X
11	E	F	Support worker carer – regular visits to the supported living home	B		X	*
12	E	F	Senior management – area of supported living sites	B		X	*
13	E	F	Senior management carer – residential supported living homes	B		X	*
14	E	M	Support worker carer – regular visits to the supported living home	B		X	*

Org: Participants were affiliated to one of five organisations: (A) Provider of residential care/nursing homes and community care; (B) Domiciliary care service; (C) Community nursing service; D Research institution project with focus on social care and technology; E Provider of residential supported living. M/F: gender. Data Collection: An X represents this participant undergoing this data collection. The * represented validation of the process map created from the data collection with other participants.

## Results

Site A implemented the lock and 49/68 (72%) staff stakeholders enrolled on and used the FRLT system, at Site B 18/18 (100%) staff stakeholders enrolled on and used the system, further to all six residents (100%). No residents at Site A were uploaded due to lack of resident capacity. Site A staff who chose not to enrol did not provide reasons.

### Pre-implementation interviews

A table of themes, codes and example evidence is available in Supplementary Appendix Table A3. The full table of evidence is available in the Supplementary File. Data suggests standard access systems across care homes, nursing homes and domiciliary care recipient's homes involve pin-pads where stakeholders enter a pre-programmed code on a number pad to gain access, or lock boxes where physical keys are stored inside a box accessed again *via* pre-programmed code.

One key theme of the pre-implementation data was *concerns with the current system.* Stakeholders perceived important issues in security, safety and efficiency of the current lock/access system across social care settings, including care homes, supported living and homes of those receiving domiciliary care. Concerns encompassed serious security flaws, including security concerns due to “*key codes being shared between multiple workers [which] causes a risk*” (P1), or codes not being changed “*for at least three years*” (P2) despite high staff turnover. Further to concerns on people gaining wrongful access, worries were also raised towards residents exiting the sites unsafely, for example as “*actual codes are written on the […] doorframe above the door*” (P2) and “*residents are quite smart, they watch you. And then […] play with the codes. And sometimes they work them out*” (P1). Participants also raised concerns on inefficiency when access was not easy and quick, as “*we do a lot of driving […] when we get there we want to get in […] get on*” (P6). The role of those providing care is busy and challenging, and time wasted can increase pressure for staff. While staff who had one consistent work base had access to codes for pin-pads, a number of participants worked across several homes and did not have access to all the entry codes, they reported being “*left standing there [at the door] for quite a long time*” (P6) as staff inside are “*busy […] doing important things, caring for people*” (P6) and the individual awaiting access did not want to feel like they were “*harassing them by banging on the door*” (P6). Such access delays were reported for NHS staff, care service staff and other visitors without codes needing to wait at doors to be let in, sometimes more than 20 min due to high pressures and workloads on staff inside the sites. For visitors attending several sites in one day, the cumulative wait time creates challenges. Considering the timing of this study during the Covid-19 pandemic, participants also raised a number of concerns on the shared touchpoints of pin-pads and key-boxes, as “*nobody cleans it after every use*” (P3).

While perceptions of the current system were predominantly negative with regards to the security, safety and efficiency offered, the theme *benefits of the current system* included initial codes on the low cost, as “*the pin-pads don’t cost anything to maintain, […] once they’re there, they’re there, so it’s kind of more a one off cost*” (P3). Additionally, participants raised the point that staff understand the current system, “*everyone kind of knows how to work them*” (P5) whereas new technology may require learning, as people may not “*instinctively go up to it*” (P8). A further benefit to visitors having to wait for staff to allow entry, was that participants felt it provided “*a sense of autonomy*” (P5) and control, in case they were “*on the loo or in the middle of making a cup of tea*” (P5), so they could take their time to answer the door.

The pre-implementation results also showed openness and interest to FRLT as an alternative, through the theme *benefits to FRT*, seen as potentially “*much more secure*” (P4), more efficient for staff and visitor access, saving staff time, ease of NHS clinician visits and reduced waiting times at the door. Health professional participants reported “*if you could get in quicker […] you could get on to the next appointment quicker*” (P8). One participant reported FRLT would mean “*safety for residents*” (P1) and “*quicker access to restricted areas for staff*” (P1). Participants also noted it would mean one less shared surface “*which could potentially spread Covid*” (P2), as the FRLT could mean “*minimization of cross contamination*” (P4). Participants also showed interest in future use of FRLT through the theme *FRT suggestions,* where ideas around improvements and additions were presented, including adding “*a heat camera [for] somebody’s temperature*” (P3). Participants also felt the FRLT could be useful in a fire as “*a system that knows who’s in the building*” so staff are not “*running around trying to find someone who doesn’t exist*” (P3). Further ideas related to potential to log staff hours by linking the access system with “*DayForce*” (P3), which again would “*save us quite valuable time*” (P4).

While perceptions towards the proposed FRLT system were mostly positive, initial queries were raised under the theme of *concerns with FRT.* One such concern was raised through the initial code of *changes to facial features,* such as if someone “*shaved his head*” or grew a “*full beard*” (P2). Technical concerns related to “*a power cut*” (P2), or the “*WiFi*” (P3), as “*how would [we] cope if we did have a power outage*” (P4). Additional concerns were raised towards weather conditions which could impair clarity of the picture for the FRLT to recognise the person, such as “*rain or […] dark*” (P5). The logistics of using the FRLT within a care home also raised some queries, including the high volume of likely users as “*we’ve got 150 staff*” (P3), while for use in the community, cost effectiveness was a key concern, “*it’s balancing the cost […] I wouldn’t pay for it because I could get a key*” (P5). Finally, some worries were aired on privacy perceptions, with facial recognition considered “*intimacy in a sample*” (P2) and “*people think you’re spying on them*” (P3). Despite these concerns, a final overall theme of *FRT acceptability* balanced perceptions, with participants noting current devices already use FRT such as “*my phone*” (P2). Overall, participants generally felt they would be open to trying the system themselves “*without a doubt*” (P4).

Summary of issues identified in current practice around**:**

• Impaired security for care homes, supported living and domiciliary care with key-box or entry codes shared across many staff and non-staff individuals
• Impaired security with entry codes being overseen and not changed regularly, problematic with high staff turnover
• Impaired security with residents guessing codes and leaving the home when unsafe to do so
• Infection control concerns with shared surfaces
• Significant delay for visitors, NHS and healthcare professionals when visiting care homes and supported living sites, waiting to be let in by busy care staff
• Care staff burden in answering door, removing them from care for residents
• Sign in books and fire registers have GDPR issues and are inaccurate

### Post-implementation interviews

A table of themes, codes and example evidence is available in Supplementary Appendix Table A4, and the full table of evidence is available in the Supplementary File. Following six months of implementation, interviews and focus groups highlighted a number of benefits of the TouchByte facial recognition access system through the theme *benefits of FRT for residential care settings.* One significant benefit noted at Site B where residents have capacity to leave the home unaccompanied was the code of *improved customer or resident autonomy,* as staff felt the FRLT “*gives them [residents] more autonomy*” (P13) without needing “*staff presence to go and open the door*” (P13). While residents could leave unattended, they did not have keys to the property or know the codes, therefore to leave or return they needed to wait for staff to support with the door. However, following FRLT implementation, they “*can come and go alone […] it’s a benefit for the customer ease. You know, they don’t need us to open the door for them*” (P10). Staff reported “*even though there's somebody here 24 h a day. There's always a member of staff here. [FRLT] helps with their independence*” (P10). A further benefit was seen through the code of *improved efficiency for care settings,* with staff reporting FRLT is “*cost effective with time management*” (P13) as it “*frees up time for us to be doing the workload that we have rather than […] I often answer the door […] it’s a constant flow.”* (P13). Staff also reported “*getting in faster”* (P11) than when using codes, and less “*delay waiting*” at the door (P13) for those without codes. Within the *benefits of FRT for residential care settings,* staff also reported benefits for improved security, as it “*stops […] people coming in that […] would know the code […] but don’t necessarily work there*” (P8), with FRLT meaning “*security with staff that change their careers*” (P1) considering the tendency for codes not to be changes for a number of years. Finally, within *benefits*, staff noted *improved safety,* with greater accuracy of knowing “*who’s in the building*” (P10) than the current written visitors book, further to one less touchpoint for “*infection control*” (P2).

A further theme from the post-implementation data was *positive reactions,* with many comments towards the reliability of the FRLT, with one staff member reporting “*I’ve been in here virtually every day […] only once that it went red [failed to recognise] and then I waved, and it went green straight away*” (P13). Participants also reported the camera was reliable “*in the dark*” (P12) and “*with my face mask up”* (P4). The participants also felt the red/green light system was intuitive as “*green means go*” (P14). Based on the data, a further code of *widespread implementation* was noted, whereby staff commented that FRLT should be “*quite widely used in care homes and in domiciliary care as well*” (P8). Staff reported that for “*the future of […] supported living schemes […] it gives security of a building*” (P12). Participants also desired FRLT integrated throughout the inside of the home, such as for staff rooms or “*medication room*” (P2) so “*any staff can get in the staff room*” (P8). Participants demonstrated positivity towards adoption of FRLT, as “*customers love it*” (P13) at site B, and “*all the staff that work here, […] they’re all using it*” (P2). Staff reported “*I’ve got no negativity*” (P10) and suggested they “*would continue to use it”* (P8).

**Figure 2:**
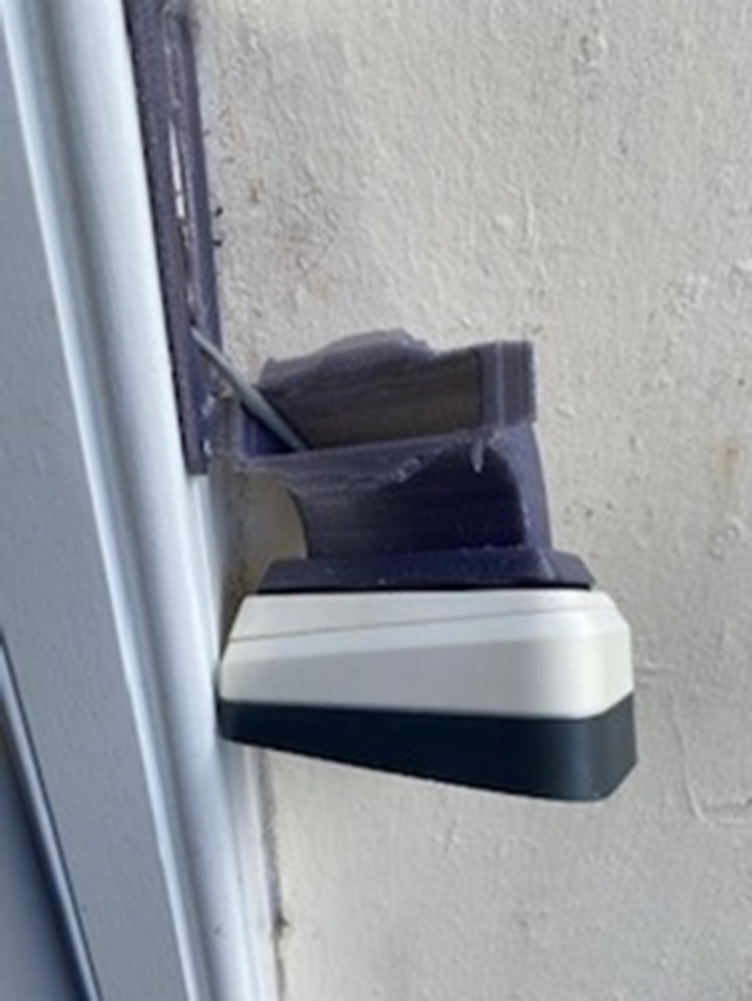
Robustness issues noted for wall mounting, which was broken on two occasions.

Despite the benefits and positivity, the theme of *barriers encountered* demonstrated some of the challenges or worries raised during the implementation period. One challenge was robustness of the camera itself, which “*a little old lady […] ripped off the wall*” (P12). Staff felt the original design “*wasn’t robust enough*” (P13) (example Figure 2). One staff member also worried about long-term “*workload, to be uploading and removing and scheduling visitors*” (P9). Participants also continued to worry about “*a power cut, would it unlock?”* (P12), although this issue wasn't encountered during the study period. Further issues were identified through the code of *negative reactions,* with participants reporting “*I can see there being a benefit. It just needs […] tweaking slightly to make it more user friendly*” (P4). These negative reactions related mostly to when people had encountered issues in their face being recognised, causing them “*to give up”* (P4). Staff also reported *problems encountered* including a supported living customer struggling with understanding positioning for his face to be recognised, “*he’s putting his face right up to it […] getting too close*” (P14). Some staff reported colleagues were not using the FRLT as “*they’re very old school, stuck in their ways*” (P4), both of which suggest further training may be required for wider successful use within adoption sites. Beyond user issues, the weather also caused challenges, with “*glare from the sun*” (P11), participants suggested a “*little awning […] to shield some sunlight*” (P2) in response.

A final theme seen within the data was *suggested improvements,* where staff provided ideas and comments on how to further improve the usefulness of the FRLT to the care setting. Some of these suggestions related to *accessibility considerations,* including “*audio feedback*” (P8) for people with sight impairments who couldn't see the lights, or were “*colour blind*” (P8). Participants suggested integration with numerous pre-existing systems, including to “*link to DayForce [staff clock-on app]”* (P2) which “*would get carers massively on side*” (P2), saving them time on logging their hours on the smartphone app. This was echoed by other staff members who felt it would be “*massively beneficial*” (P4). Participants suggested an additional camera “*on the way out*” (P11) for “*fire registers*” (P10).

Summary of issues raised included:

• Improved autonomy for supported living residents to leave and enter when desired
• Ease of access for staff not remembering many access codes
• Mainly faster access for staff, residents and visitors, less delays
• Reduced staff burden and staff time saving in answering door and leaving important tasks
• Improved perceived security with no code sharing
• Improved perceived infection control with one less shared surface
• Robustness requires improvement
• Some frustration with reliability/speed of system leading some staff to avoid use
• Some issues in sunlight/weather impacting recognition of faces
• Accessibility considerations for colour blind, potential to add audio feedback to aid in positioning face to camera

### Process mapping

**Figure 3:**
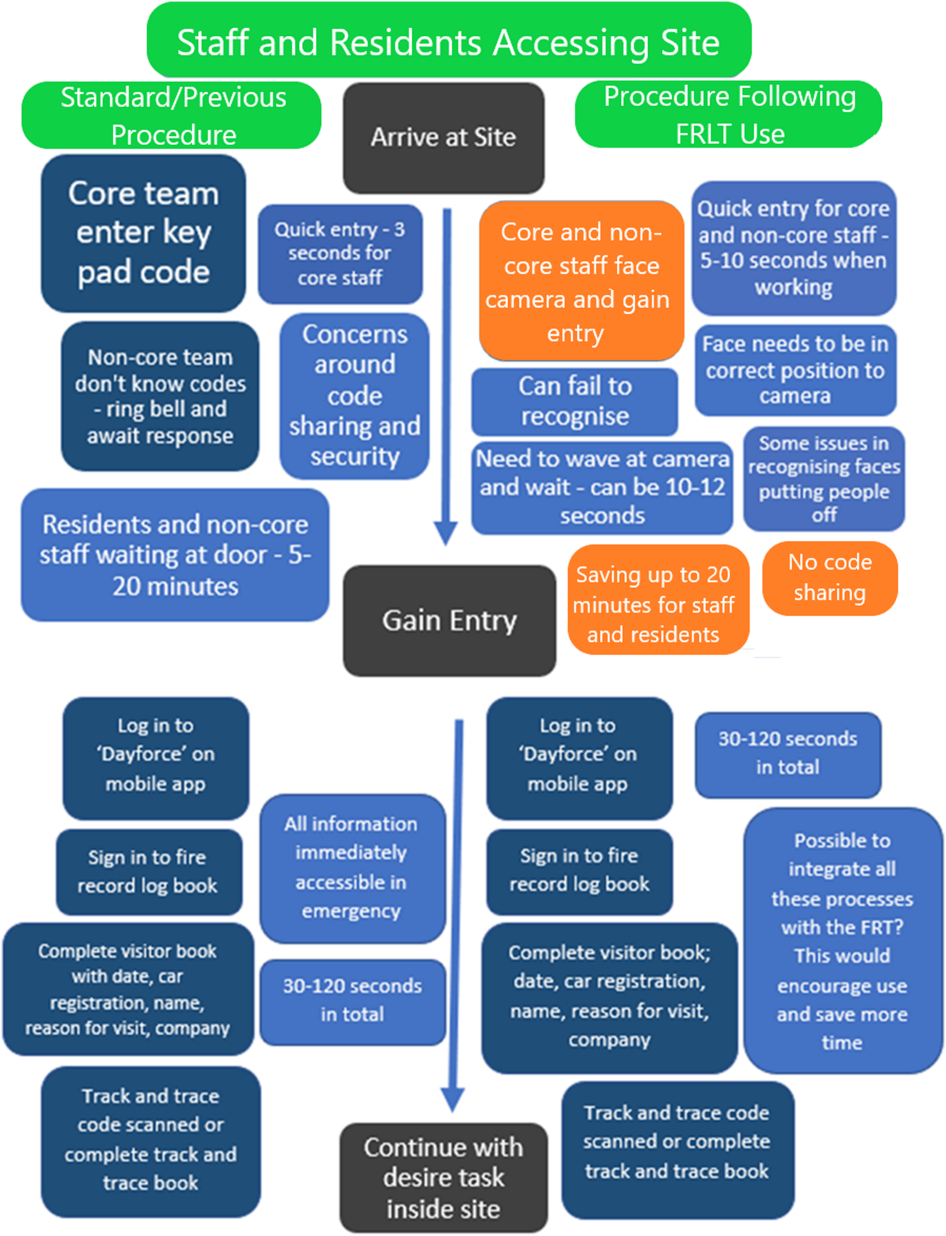
Process map for accessing residential care sites before FRLT implementation (previous procedure – left of the figure) and following FRLT implementation (right of the figure) for *staff and site residents.*

The process mapping exercise was undertaken by five participants (3 core care staff, 2 visiting professionals), and also verified with four staff participants who read the map and confirmed accuracy of the results from their perception. The pre-implementation process map (Figure 3) highlighted some areas of inefficiency and concerns, particularly for staff that are not core to the site and do not know the pin codes for all care homes or sites they visit, and other visitors and health professionals. These visitors reported delays of up to 20 min. Further issues were noted with regard to residents with independence to leave the site also needing to wait outside to be allowed in. The delays in admission related to the busyness of the care teams inside the properties.

Dark blue boxes indicate key activities, light blue boxes provide additional comments and information, orange boxes indicate a potential positive change in response to FRT implementation.

Dark blue boxes indicate key activities, light blue boxes provide additional comments and information, orange boxes indicate a potential positive change in response to FRT implementation.

**Figure 4:**
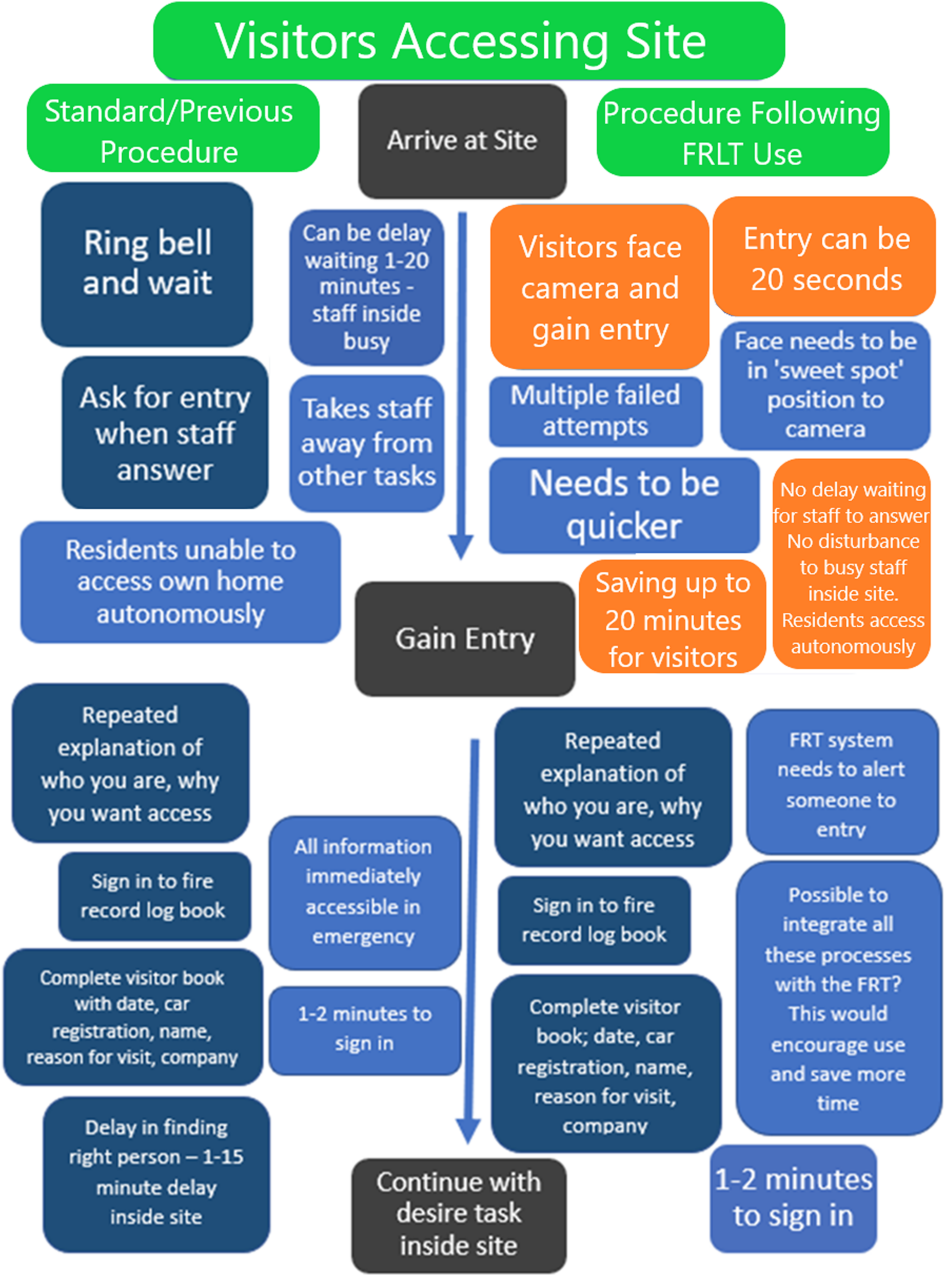
Process map for accessing residential care sites before FRLT implementation (previous procedure – left of figure) and following FRLT implementation (right of figure) for *visitors.*

The post-implementation process maps (Figures 3, 4) identified some time savings for core staff, visiting staff and other visitors, who saved up to 20 min of waiting for a door to be answered by gaining direct access with the FRLT. The staff identified during process mapping that residents could gain immediate access and no longer had to wait, enhancing autonomy. Visitors (and staff commenting on the process for visitors) noted during process mapping however that an alert to staff of their arrival would be advantageous and staff noted that integration with other systems would further enhance usefulness.

### Follow up at 6 months

In follow-up contact with the two sites, we were informed that Site B had made a positive procurement decision to pay for continued use of the FRLT and discuss wider roll out within their company. Site A also chose to keep the FRLT installed, but has not yet committed to a procurement decision and wider implementation due to current financial pressures.

### Follow up with the developers at 12 months

The product developers, TouchByte, were provided with the results of this study with the aim of further product development informed by end-user feedback. The developers were then contacted following 12 months to provide the opportunity for an update on any technology amendments in relation to the study results. The response below was received from the developers:

*Throughout the trial period at the two sites, TouchByte has continuously worked with users to enhance the system based on their feedback. The formal findings of the academic research have been invaluable in this respect as they form structured input from a range of users that has been rigorously examined and collated. This has been very valuable in supporting and supplementing the ad hoc feedback gathered by the TouchByte team throughout the trial period. Since the original installation of Facentry (company name for FRLT) at the two sites we have made the system more adaptable to suit a wide range of different buildings and positions. We have:*

• Developed a modular 30°, 45° and 60° angle mounting bracket design, allowing camera positions to be optimised for the location when installed.
• Trialled 3D prints of these designs at a number of sites.
• Optimised the designs based on in service experience (e.g., physical breakages).

*Based on feedback from day-to-day users regarding response times for the system, we have made a number of developments and changes. We have:*

• *Trialled and implemented alternative proximity sensors which detect the approach of a user from further away*.
• *Further developed the camera module electronics to increase the sensitivity of the proximity sensor adjustment*.
• *Experimented with and adjusted the parameters that are used alongside facial recognition to enhance system response time*.

*Together these updates have helped reduce the system response times, improve the detection of faces so that the user does not have to be in a “sweet spot”, and reduced the number of unsuccessful access attempts for authorised users*.

*We have also developed a number of the suggested improvements:*

• *We have developed a system called “NotifyMe” which can alert staff members to the arrival of a particular person via the Facentry system. NotifyMe currently uses MS Teams or email to provide the alert*.
• *We have also now developed FaceOS, the management platform that controls enrolment, scheduling and reporting. This has been introduced at both sites*.
• *As part of FaceOS we have developed an emergency roll call system accessible via mobile phone. Using this, a controller of the system on a site could conduct an emergency roll call and, if a particular person is not there, could immediately call their mobile phone direct from the register to check that they are safe*.
• *We have developed a version of the camera module that has three status indicator lights rather than one. This allows us to use pictograms in addition to the white, green and red lights, thus addressing the colour blindness issue raised*.

## Discussion

This study has provided insight into the security and access experience in UK social care settings and how FRLT may help. Stakeholders from care homes, nursing homes and domiciliary care cited various problems with the current systems that generally involve pin-pads and lock boxes, both accessed *via* pre-programmed codes. The limitations included safety issues from code sharing, codes being over seen, shared high-touch surfaces, delayed access for professionals who visit multiple sites, and care provider burden from managing their care workload and access system. Currently paper-based sign-in/record systems are open for inaccuracies and GDPR issues, which become a dangerous limitation with regards to fires or other incidents. Innovations such as FRLT are needed.

Over six months good adoption rates for FRLT were seen at our two centres with most staff using it. Most of the time access was quicker following the implementation of FRLT, easing entry for health and care professionals and particularly those without access codes who visit multiple sites. Staff felt there was less time spent needing to leave their care duties to attend to the door. Security was thought to be improved through reduced code sharing and some felt infection control considerations of the touchless device was a benefit, although this view was not shared by all participants, with one noting the number of other shared surfaces to gain entry.

Considering the importance of perceived security in choice of care homes for resident relatives (13), this study has implications for real-world practice. Installation of FRLT to enhance security and access may positively impact selection decisions, although further research with family members is required.

As noted in previous work, security involves a careful balance between autonomy and safety (11), with this study suggesting FRLT improved autonomy of residents at Site B. While pin-pad codes are generally only known by regular staff, the FRLT allows the flexibility of uploading residents' face images who have autonomy to leave the site unaided, while still maintaining safety for those without capacity to leave unaided, who would not be uploaded to the system. This has potential implications for resident wellbeing, should they have greater access to outdoor spaces and activities (15, 30). Flexible and enhanced autonomy for social care residents is a major potential benefit of this technology.

The study also noted some limitations to the FRLT innovation and required improvements. One such limitations was robustness of the wall-mounting, with two cases of the device becoming broken, once by a service user trying to re-angle the camera and once by a passer-by believed to have dementia. These perceived concerns were far less prevalent than the noted benefits however. Some negative responses suggested using the FRLT was actually slower than standard access, but again these cases were in the minority and may be a result of the early stage implementation and required improvements to system reliability. As seen in the post-implementation process map, this caused some frustration and delay for participants affected.

Further improvements were noted in the “suggested improvements” theme, with accessibility considerations a key concern, with potential to include audio or positioning feedback in future iterations. Considering the limitations of the current paper-based sign-in method, further improvements suggested were integration with the fire register and other systems, to enhance usefulness further and generate additional buy-in from stakeholders. Further research could explore perceptions of stakeholders towards integration of health care services with the FRLT, such as health prospects through aging and wellness programs (31).

Our results suggested some minor concerns around privacy with uploading face images, this echoes the concerns highlighted by Van Natta et al. (23). Although most staff were comfortable with uploading their face images and using the system, future research may wish to explore privacy concerns more directly, and particularly in light of integration with the staff clock in/out system, to assess any monitoring concerns.

## Strengths and limitations

One strength of this study is the novelty, with limited work to date exploring the concept of security and access in social care settings, and distinct lack of innovations in this area to date. This study involved exploration of a novel technology in two different residential care settings, one care home with people with less capacity and one assisted living facility where residents have more autonomy, suggesting acceptability and willingness/usefulness for adoption in both settings. The real-world implementations and evaluations means our results have good ecological validity. However, a limitation is that only two sites participated, limiting generalisability somewhat and leaving scope for further research in this area. Although we have data suggesting usefulness in domiciliary care contexts, we have not yet been able to study this directly. Further implementations would be required to assess generalisability across further care homes and supported living settings. We were also unable to explore perceptions of visiting friends or family, due to limited visitations during the study period in response to Covid-19. Therefore future research may also explore the impact of easier access for visitors to residents. Finally, a further limitation is a lack of direct exploration of ethical and regulatory considerations, while limited concerns were displayed by stakeholders in this study. This may demonstrate lack of awareness of potential pitfalls and need for further education. One such consideration when thinking of FRLT for care homes is informed consent to continued use (32) particularly when the intended users may experience fluctuations in capacity.

## Conclusion

This work has identified previously understudied concerns on security and access in social care settings, which warrant further exploration and research. Our findings demonstrate scope for innovation and improvements in this area. Our results suggest potential usefulness of FRLT to this regard, particularly if systems could integrate with pre-existing systems and be cost-effective. Key areas of potential improvement to enhance usefulness further would be integration of the camera with staff clock-on system, visitor book and fire system, to ensure accurate logs of who is at work or in the building. In order to adoption to be realised, improvements to reliability and robustness are also needed to ensure optimisation for social care settings. This report supports the potential usefulness of FRLT lock systems for implementation across social care settings, including supported living and care home sites, due to the potential for improved resident autonomy, staff use of time, ease of access, security of the site and infection prevention.

## Data Availability

The anonymised raw data supporting the conclusions of this article will be made available by the authors, without undue reservation.
