# Supplementary material for: Facial recognition lock technology for social care settings: A qualitative evaluation of implementation of facial recognition locks at two residential care sites
Source: Front Digit Health. 2023 Mar 3;5:1066327. doi: 10.3389/fdgth.2023.1066327 (PMC10020502; doi:10.3389/fdgth.2023.1066327)
Supplement: Supplementary file 1 [file Table1.docx]

**Supplementary File: Tables of Evidence**

**Table 1: Full table of evidence from pre-implementation interviews**

| **Theme (n)** | **Grouped codes (n)** | **Initial codes (n)** | **Evidence** |
| --- | --- | --- | --- |
| Concerns with current system (92) | Current system – security concerns and inefficiency for community care (16) | Key box issues (4), Control undesirable access (5), Ease of carer/NHS access considering mobility or hearing impairments and code sharing issues (7), | “And the one thing that commonly I had to pick up on was that they would put the code in, so you pull down the little panel, I know what you mean, yeah, put the code in, like click the sort of catch down, open it up, take the key out, some people will then leave it open. But what they're doing is also leaving the code by they just they can check what code was, or some people will push it up. But then again, they will leave the code and slide it up. And I always used to say you don't know what's going to happen whilst you're in, you know, working with your client, he will come by so I always make make a point that you should shut it, change the lock again, like lock it and change the code and put the thing back up and then unlock it when you want to return the key. Yeah.” (P2)  “He has a carer visit and they've got like a lockbox on the door on the wall. I mean, if they have a little camera installed, as long as obviously the weatherproof and sort of robust. Yeah then the carer would obviously, just be able to go up to the door and open without having keys hanging around. You know, although they're quite strong, it wouldn't take much for somebody to just break it open.” (P4)  “I never really trust them. And my mom was saying about getting one for her bungalow. And I said, just give me a spare key. It's much safer. I don't trust it like that. But with a camera, you know, you can't gain access unless you've got the face” (P4)  “it's a lot down to mental capacity, what that resident wants and what they consent to. So we have a person in a in a block of flats, it's only two or three storeys, not very high. But what a state, and they've got a daughter, who spit like a zombie, quite honestly, seriously, uh, you know, I don't use the term lightly. And they have a lot of people coming and going, because people get to know that there were two women in a house. And they, they know that there's a certain supply of food and, and medications. And, you know, sexual activity has been witnessed, do you know, so it's, they've got no access, there is outside the flats, that's normally there's a trade button. That's a big issue, anyone can press a trade button and get straight here. And then they've got nothing on that actual door to stop anyone going in, but that's how they live,” (P6)  “there are certain people that control that situation that they access the house, and they sort of dictate what carers are going in three times a day, I think, in that house, and we're now doing welfare checks every week. You know, Doctor has said that they've got capacity. So if someone's got the capacity to make unwise decisions, and but, you know, just to sort of see that the daughter is mobile, you know, her teeth are rotten. She looks like she's pregnant. She isn't I God only knows what's going on there. But she doesn't consent to anything, that her mother is losing weight, this is our patient and wait, if she doesn't consent to be weighed, she won't have a doctor's visit. So we did get one as an emergency recently. But so as an ongoing situation, and the carers say things just disappear from the house. In a we order, you know, even creams for skin and things like that pads, you know, people take them and they sell them on eBay. Yeah, people need money for things. So that's in a small block of flats, which is otherwise very well kept. Those residents, particularly our, you know, live in a very sort of feral way. And you know, nobody seems to be able to do anything about it.” (P6)  “So the other thing? Of course they do is he got someone on the door trying to let people in or they forgotten the keypad number, or? ” (p3)  “But I'm just thinking, you know, part of the problem there is that some of the some of our nurses, what if you're an age when and when a woman is not pregnant, they want to get pregnant, and they're worried about taking so I don't think we can breed prescriptive by that. But I suspect if there was somebody that was high risk, you can use that to say, actually, this client is high risk, it only needs to be certain people go in there, and then you can control that.” (P3)  “I never really trust them. And my mom was saying about getting one for her bungalow. And I said, just give me a spare key. It's much safer. I don't trust it like that. But with a camera, you know, you can't gain access unless you've got the face” (P4)  “Not Generally, the problems that we would encounter would mostly be at private homes, where the homeowner is probably got reduced mobility. And you don't want them rushing to the door in their terrible slippers that they've set up because they can't get the Bonanza. And they've loved all their nice rugs on the floor that they're tripping over and the cats and so all they just simply are deaf and they can't hear us” (P6)  “And so, yeah, that's the difficulty really, because we're not allowed to wear anything over our arm. So we've got to just have our uniform off, which is a pretty cold and wet often and Western on the doorstep thinking, Are they dead? You know, what's happening in there? Have they deteriorated to an extent they can't answer the door? All these things really? Or are they just really slow? Yeah. Which we understand, of course, and allow for but yeah, they're the sort of typical practice as you'd appreciate” (P6)  “So in Council properties, we can apply for keysafe to be put up. And also, as well as our service people have carers, and they have volunteer support to do shopping and things like that. So there's a whole raft of people that are going in, people might be walking their dog, if you've got one. And yeah, so we're just one of many people who need access to the house. And, yeah, so we have a lot of key saves. In fact, I instigated a key safe. I'm like the key safe monitor in our team because I was fed up of turning up places. And even when you you know, the people are expecting you. The Doors aren't open, and it's such a waste of our time really is and so I do a list which you're not supposed to do a key saves. And every month I we knew that list and people take a shot of it on their phone. So that they've got it. But just last week, we amalgamated with three other teams, okay, within our area. So they've now tried to put us all in one room. So and also they're very overstretched, and we're not so stretched at the moment. So we are seeing a lot of patients from other teams that we don't know. And they often omit to tell us the details of Oh, it's rounded back, it's under under the coal bunker.” (P6)  “we do a lot of driving. So of course, when we get there, we want to be able to get in, you just want to get, get on with what you're there for.” (P6)  “Of course, the worst case safes are those ones where you have to remember the code to get in it. And then they've got a sort of removable section and they're tiny, when you're trying to shove the keys in. And then you've got to also re enter the code before leaving. And if you're not, if it's not one of your regular patients, and you're doing it for another team, then you're you're having to get your phone out to then access the document where you've kept your key sec numbers, if you've got it, but if it's for another team, and they haven't given you their whatever, and then and if your phone is activated by fingerprint, then you take your gloves off, and you're having to touch your own phone or a work phone to do that.” (P6)  “Because you know, one of the other teams that we've just joined with, I worked there last year for six months. So I know a lot of their patients and I did one of my key code lists. This is a bugbear of mine. It's quite interesting that Jeremy mentioned excellent. I'm a bit of a pattern like that. And you know, I just can't waste time. And then we've got two other big teams that we've now joined with. And if they don't put the information on the on the patient's notes then and because I can't act, we have a GPS system and we have our system. It used to be the same and now it's not. So we have a system called Rio r IO. And the doctors are still using a system called system one. So if those patients aren't registered with my doctors, my surgery I cannot access their medical records. To find out anything, let alone How to get in there in the first place.” (P6)  “Because keys getting lost or the key codes being shared between multiple workers causes a risk to that person within the community” (P1) |
|  | Current system -security concerns for care homes (12) | Security issues with codes (4), Safety of residents (6), Poor GDPR (2) | “I don't know if you've seen it yourself, but in the past, historically, a lot of places and it's not just called cat and lots of different cat providers, you would see the actual codes written on the sort of doorframe above the door. So any member of staff or coming along could just look up and read the code and put it in. But that was going on the assumption that every client and residents isn't going to be able to see.” (P2)  “And the other with that with codes as well, if some services, they'll have the same code. So ones that I know, they've had the code, they've been saying all of the external doors for at least three years.” (P2)  “do they get do those numbers get changed often enough?” (P3)  “you know, that's especially his unit and you certainly don't want to let people out of there because that can be dangerous, etc, etc.” (P3)  “But, you know, the the codes are shared and used very widely and vary quite a few times the day that if there is a resident that you know, is overseeing, overseeing, overseeing it won't take them along, even though they do have an impaired cognition to be able to work that out. So for me, it's around our residents safety.” (P1)  “And, you know, not many people will attempt to hide that pin, you know, they just push it because that's, it's just, it's just so instinctual, isn't it? You don't think about it, it's how you get in and out. And they're doing it and maybe, you know, maybe hundreds of times a day, they just press the buttons 0000 classic one or 1111, whatever, you know, you can you there's no attempt to hide it. And you can't blame them for that, because they're busy, and they do it every day, and they're not gonna make the effort to cover it up. So I definitely think there's a security, you know, a security risk there. And whether they change them or not, I'm not really sure. But I would imagine that people who don't change very often, you know, it's like a pin number, isn't it? If you if you know it, you know it and you know, you're gonna get access. So why would you change it? Because that's just going to be an inconvenience to you and the rest of your staff? So, yeah, I can understand why they wouldn't, they wouldn't change it” (P8)  “Because the codes obviously need to be changed to ensure the safety and security for the residents and the staff. When staff move or soft change, or if a visitor over here is one you never know or overseas one.” (P1)  “Some of our residents quite smart, they watch you. And then they're going play with the plants. And sometimes they work them out by fluke.” (P1)  “So the biggest issues for me with Reliant reliance on a keypad system is safety with regards to staff coming, you know, staff leaving employment, and the systems needing to be changed in in a timely manner. Residents that don't have capacity, but work the code out, you know, the risk of absconding I don't like because you put controls in place to rescue mitigate that risk as much as possible.” (P1)  “But, you know, the the codes are shared and used very widely and vary quite a few times the day that if there is a resident that you know, is overseeing, overseeing, overseeing it won't take them along, even though they do have an impaired cognition to be able to work that out. So for me, it's around our residents safety.” (P1)  “I know that a couple of times. residents have tried to get out. And because they've been pressing the button so many times they've had to change the code or add numbers to the codes” (P9)  “it will be able to allow us to have a login, for example, a GDPR compliant login of who isn't is isn't isn't in the building, because I hate visitor books for that reason” (P1)  “So for me, I would like to see to see us be able to work around complying more with the visitor login and the name call of who's in the building, instead of having a book or a list that's actually readily available for all and sundry to see. Yeah, even though we have got the GDPR. You know, you might leave out the blank. But actually, if someone goes through it, for example, if it's not overseen there is there is a risk. So to me, I feel that we'd be able to improve our GDPR around around that” (P1) |
|  | Current system – safety and security concerns for care homes (14) | Covid spread (10), Code known by many users (3), Broken systems (1) | “Because you know, we don't have to touch anything. So where there are sort of the key codes, panels, you know, people still touch, which potentially could spread COVID” (P2)  “How often do they cleaned, and if it's not every visitor, then it's not enough, given given COVID right now.” (P3)  “So if you've got if you got a PIN code on your on your door, and nobody cleans after every use, then actually it's just a point of entry. And so when we had the second wave of COVID, last November, we didn't know what hit us. He just came at us thick and fast. And it seemed to be just coming through the windows, you know, I mean, it was just it was it that shook up so we lost quite a lot of people again” (P3)  “So we all use the same pen. And you have to go to the loo and wash your hands when you when you go in the door. And then you open the bathroom door? Well Hang on a minute, you're back to where you were. So then you sign in, and then you put hand sanitizer, and get it in order. And yeah. Yeah, interesting.” (P3)  “So bear in mind, we got properties, we've got people administrating drugs, you've got people minister writing care, we've got a learning and development Academy we've got. And we've got people out in the community, the risk, the wave band of risks that can be involved is huge.” (P3)  “So yeah, and again, you know, nurses have nurses going to work across two or three homes,” (P3)  “Yeah, in the same way, we've got support offices as well, how'd you get in and out of support office, you have to sign in a book.” (P3)  “Equally, I suppose. I just think then, you know, if you've got a firebreak and you've got an emergency system, actually, you do not want to be waiting to press buttons. You want to be able to get out that no, absolutely.” (P3)  “Yeah. With with infection prevention and control. This is quite simple. We would we use, we, we use a spray, we will clean that we clean the key, we clean the bathrooms, we popped it back. There's no risk. No, no. Again, I can imagine that if people are.... some other organizations who don't provide PPE and aren't as robust as we are around infection control, then of course, it's going to be a risk” (P5)  “I can imagine with larger organizations who who don't take as much care as we do in sharing information and send different carers on different days, different people can imagine that being an awful lot of people with, with with knowledge of somebody's keypad who don't need who shouldn't have it, or don't really need it. That's a really good point. security issues about the more the more people know, the key code the the more vulnerable they become.” (P5)  “Yeah, obviously, you got to the key pads, I think they get a half hourly clean. But yeah, there is a worry about obviously, cross contamination, etc, etc. Because people's fingers go everywhere without even people knowing about it.” (P4)  “Yeah, I mean, I imagine so I mean, obviously working in the kitchen, you're very, I've got a level three food hygenie certificate, so I'm very genned up on the cross contamination issues. So anything that can be transferred by hands, you know, is obviously going to be an issue with a keypad system. There's physical touch.” (P4)  “There push buttons, too, sometimes they can get damaged quite easily, especially when you got dementia client, really trying to get out and smashing the keypads up? And so but yeah, apart from that, they work quite well.” (P4)  “Because it's very easy to pass on a number, isn't it?” (P5)  “obviously with hand sanitizing washing hands, how many people are touching those? At the moment? Because we will hope that we've all got good standards of what could be a lot of people. Do you know if they're cleaned?” (P9) |
|  | Current system – Inefficiency concerns for care homes (50) | Delay in entering (11), Staff don’t know codes (6), Having to learn codes (6), Doorbell or card failures (3), Hands full (2), Institutional (1), Time costly relaying codes to agency (2), Rust (1), Time lag (2), NHS visitors inefficient access (2), Visitors stuck inside (4), Staff too busy to attend door (4), lost key fobs (1), Onerous (1), Inaccurate visitor log (3), Visitor feels burdensome (1) | “So currently that I enter, is that I go in, and I go to reception and speak to the administrator, kind of just start when you go in how to get in through the door. So that the front door currently it goes into sort of like a little like a glass walkway. So that that areas open. And then there's sort of double doors that goes into reception area, which is key coded, right. But I don't get given the key code, because I've worked different services. So right, give me the key code someday, the [site], I don't know that one. I just sort of like waiting for the window, and then the admin lady will then come and open the door. And then I sign in the same as any other visitor. The homes where the staff know me, they will sort of let me you know, as soon as they see me the same as they would a permanent member of staff from that service. But there are some services where there's new staff. And because I'm not a member of their team, they're quite unsure who I am. So sometimes I do sort of stand around outside waiting for them to find somebody who knows me and, you know, doing all the right sort of security checks, but sometimes it can be slightly inconvenient because we COVID and things sometimes I've been sort of stood out selves for about 20 minutes. Yeah.” (P2)  “I sign in the same as any other visitor.” (P2)  “So you know, it the staff who come to the door don't know me. They won't just let me in on the one thing from l&d.” (P2)  “Quite often people sort of say, Oh, can you just wait there, I'll go get the manager or somebody like that somebody, you know, to deal with you” (P2)  “ And if it's cold and wet, you don't really want to sort of be stood outside the door waiting.” (P2)  “in occasions where that's been quite inconvenient for me a long time.” (P2)  “So, but they’ll key, enough to learn, learn the code” (P3)  “nobody's going to know I've been in my lunch break.” (P3)  “I remember hearing a story about a lady who was on a special diet and maybe mixing two stories, same effect. And so they came to deliver drugs. And she didn't answer they're also delivering. And I wasn't he she was this. This person was on medication. And they were in hospital again a week later. And it's why aren't you taking the medication? They never delivered it? Why don't deliver it? I don't I say find the pharmacies and we tried to do it, we can get an answer. Okay, what was wrong? What was wrong with the battery was gone in the in the doorbell. That was all it was. So for the sake of a doorbell, we've now got three sides of a bill for looking at somebody else. We need to be able to do deal with stuff like that. And the lady who was on on a special diet to build a health and strength, but actually couldn't, didn't have enough strength to go to the, to the shop and pick him up and bring him back suddenly, just crazy stuff.” (P3)  “When the carriers are going into the laundry, and the carrier loads of stuff, they've got to put it on the floor, then which creates trip hazards, and they're having to code and you know, whereas they could just go up to it show their face, and it'll allow them entry straight in. So they're the things I've sort of identified just by sort of sat around thinking about it, because we've obviously known about it for a couple of weeks now. And yeah, being a bit of a tech geek myself, I've thought of all the benefits, you know, of, of the face recognition system.” (P4)  “The other thing is as well that I feel that the keypad system can look quite institutionalized. Yes. Yeah, that's one thing as a care home we want to come away from we want to do a, you know, obviously keep it as homely looking as possible, where if you've got keypads and stuff like that, it does create that industrialists, that kind of view.” (P4)  “The trouble that we do find is when you get agency staff, etc. You've then gotta then obviously relay all the codes to them. And they can forget. And yeah, it can be very sort of time consuming as well. Yeah.” (P4)  “older systems can get rusty, sometimes. People, we haven't had anybody forget the key key code on or I guess that might be an issue.” (P5)  “But I think you know, off the top of my head, gaining entry, exit, you're looking at about at least just for myself, 10 codes, with different numbers as well.” (P4)  “I just have to keep remembering the code. Nah you've got to remember, like 100 different codes?” (P4)  “Obviously, your keypad gets broken and they change the code and then you've got to try and remember that all over again. Yeah.” (P4)  “sort of little glitches, sometimes if you input the code wrong three times, you've then got to wait for a 32nd period, before you can then retry the codes.” (P4)  “Only if you input the code wrong, then you've got I think it's like a 20/30 seconds where the, the light just flashes, and then you want to wait for that light to stop flashing, and then you can then input the code, whereas, you know, if you need immediate access quite quickly, that can prove to be a bit of an issue” (P4)  “So we turn up at a big care home, say, and there's usually a bell to press, and there's usually a receptionist in the big care homes, and then we have to sign a book in and then we usually have to press another button to enter. And then we're in. And also internally, it's quite confusing because we've got people with dementia and people who might be prone to wandering outside. But there's a lot of code inside the building as well which is always a bit of a nightmare because she's, you know, they change them and then you're stuck down a courage to get out.” (P6)  “ Well, I suppose why not booked to attend they know to expect us our appointments are usually visits are usually in the mornings if you can, but we don't have appointments. Unless there was something like a specific injection to give with, but even there within usually a two hour time frame, so we don't have time to appointments. Okay? Although people try and tell us we're late on time, we, you know, we just can't because we might go to a house and find someone behind the door, and then we've got to wait for an ambulance. And then they don't come because there's a nurse in attendance. So, you know, it can easily unravel very quickly. We don't make appointments. So yes, we do often have to wait. But there's usually somebody buzzing around. So they see us standing there in our fluffy apron and our little gloves, and they come and let it in coming at us.” (P6)  “ if you're a visitor like myself, I'd be classed as a visitor. So I'm not necessarily going to be given the code for all of our homes just because of GDPR, privacy, all that sort of stuff. So I would probably have to ring a bell and wait for an answer or knock on a door and wait for an answer. Which is great if it's sunny. But if it's raining, you don't want to be standing outside waiting for ages” (P7)  “our care hidden staff and managing so busy, they haven't got time to be waiting on the door. receptionists are often expected to open the doors, but they're busy as well. They don't have time to open the door. They're closed door for everyone that coming in and out. So we Yeah, sometimes you have to wait. And that can be you know, as long as it takes. There's something going on in the home that hasn't been expected which can happen is you're going to have to wait longer. So yeah, that can be a little bit inconvenient. But that's not the fault of the staff.” (P7)  “Yeah, lots of different people going lots of different places you can imagine codes and especially for a new carers that has no real experience in care, it can be quite daunting for them. And then trying to remember the code and tried to speak a lot of key safes are actually quite difficult to work, opening them because they're supposed to be.” (P7)  “they might we have the key fobs. And again, you can quite often forget the key code. You can the weather might receive them and you can't get into there as nightmare. You might get a new carer that doesn't really understand them. And it actually can be very difficult. So yes, I could potentially see that being great, but it would really be I think up to the to the client really and maybe their family members to say yes or no” (P7)  “So the current system when you enter the home as you go into an initial like a door conservatory doorway, you have to put in key code to enter the reception area where you sign in, and then you need a further key code to be able to get into the residential areas of the home. So once you're in the main home, there are key paths in line with IPC. So first, Seuss rooms, laundry rooms, for example. Domestic storage areas, all keypad, all different codes, all ridiculously confusing when you work across 16 care homes. And each home has about eight or nine different codes.” (P1)  “from a selfish point of view, not from a research point of view, no, because if you're, you know, we're all busy. And, you know, as much as you can appreciate that the things going on in the care homes that you know, there are, they're important, and they're a queue. And they Yeah, that's, that's obviously whether rightly what their priorities are. But yeah, it can be frustrating as someone who's got to maybe get around a few homes in a day, if you've got lots of different appointments, if they will get kind of slowed down and backed up, that can definitely be a problem.” (P8)  “So normally, there would be somebody at reception, and you tell them who you are, who you're coming to see what you're there for? Where you from shedding the badge, and then they'd get you to sign in, in a book for your registration, number down your name, where you're from, what time you've come in, and then you have to do that on the way out as well. And clock yourself out. Which is quite an onerous process really” (P8)  “ signing in is it's not really 100% compliant. But yeah, signing out, I think, you know, you've had you mean, you want to get on to your next appointment, and you just forget, like, you just forget, and it's hard to just think, Oh, yeah. So it's not a conscious thing. Like, oh, I couldn't be bothered to do that. But I think you just need just to get to the next place. And yeah, I've definitely definitely forgotten to sign out for places and I don't really think anyone keeps an eye on it.” (P8)  “we did try and pre arrange meetings with care homes, you know, ringing managers saying that we'd be there at a certain time to come and deliver them the device. And it was difficult. Yeah, we're quite often left on the doorstep and you kind of ring the doorbell or you knock on the door if there wasn't a doorbell. But that is a you know, they're really, really busy. So quite often, you're left standing there for quite a long time. And then you're kind of left wondering whether anyone's heard you, or whether the doorbells even working. Quite often we'd stand there and think, I wonder if that doorbell is working. And then you obviously try knocking but you don't, you don't want to be too intrusive. Like, you don't want to feel like you're kind of harassing whoever's inside, if they're, if they're busy, you know, doing important things caring for people, you don't want to be kind of harassing them by banging on the door or ringing the doorbell, you know, 15 times,” (P8)  “ Yeah, absolutely. So even with that kind of pre permission to go in at a certain time, on a certain day, it was still difficult to gain access, like you say, purely because there's, they're just a busy and, you know, you know, games are unpredictable, and people are unpredictable. So you never really know what's going to happen on a day or a certain date a certain time. And yeah, they always busy, so they can't always come to the door straight away. And yeah, you read the left standing outside, or someone does typically normally answer the door, eventually. But typically, you know, more or less of the person that you're looking for is often busy and able to see you at that time. So yeah, lots of kind of hanging around. And, and just yeah, be patient really. Okay.” (P8)  “Also, the other element that I don't like is, we do have stuff that move between sites and agency that come in, through agency staff needing to access kitchen, cleaning, sluice rooms, infection testing, the key codes put in what I call an unwarranted barrier to accessing them. And it means that they have to go off and find so make him go off and they walk around until they find someone else to try and get it. So it causes delays.” (P1)  “And there's key codes either side, and I got stuck in the middle. So you had to ring the reception and say, I'm stuck in side and don’t know the code.” (P1)  “Because that problem with the old, the current system that they've got now is quite often, if you don't know the PIN code, you can, I've been trapped in many, many cases where you just can't get out. Because you don't know the pin number. And quite often the doors are, you know, they are locked. So to actually get back out again, it's not just about coming in, it's actually as a visitor getting back out again. And then you have to get involved with somebody to let you out which you're then presenting more of a burden on them. So yeah, in terms of being able to get out if you can just, you know, show your face to a camera. That'd be fantastic.” (P8)  “Actually, it's got lots of individual weird and some of them are locked. And it's because I don't go to those particular areas very often I have to find somebody to find the key code or get someone on the other side to let me in. So I think it would be really, really valuable actually.” (P9)  “I'd say, from my point of view, I've usually got lots of stuff with me. So I've got a wheelie case, pieces, I've got a laptop, really got a bag full of my lunch stuff. So it's having enough hands or you know, you don't want to put everything down to the number come in. ” (P9)  “you think when you're there, you can get quite inconsequential. So you kind of just think, Oh, this is just a bit of a kind of like paper, fishing exercise. But actually, yeah, if there was a fire or something went wrong, where people had to evacuate, and you couldn't account for everyone that was in that? I think yeah, that's gonna lead to problems.” (P8)  “So that the need to not have a key card. sounds brilliant. So most places that I've worked with require a key card, and I either Forget it, or it doesn't work. It literally just doesn't use you then like this, and you're swiping it, and it just it just doesn't work. And it's infuriating. So that sounds brilliant” (P8) |
| Benefits of current system (14) | Current system –benefits for care homes and community (14) | Low cost (2), No Covid risk (3), Already keys in place (1), Autonomy for community care (2), No delays (1), Everyone knows current system (3), Control access (2) | “And I don't know, I may be wrong. Because I, we haven't had a single case, as I know, of, of COVID the past from a key safe to another person in social care. I know of no. I haven't seen that.” (P5)  It all depends on it will be very highly dependent on pricing care homes as well. And you've got to think that care homes, they run on very, very tight margins. And so thank you put the keypad on, that they know that they wipe after every entry and exit. That's cost them 25 pounds. And it's there forever. And it's protected, isn't it? Isn't? It isn't dependent on weather and everything else, then they're going to have the keypad? Yeah,” (P5)  “I guess the pin pads, they don't cost anything to maintain, do they once they're there, they're there. So it's kind of more of a one off cost […] And it’ll be cheaper than the cameras I guess” (P3)  “They want someone to ring the doorbell they want to go and answer it themselves. They want to know who's there” (P5)  “And it's almost that perhaps sense of autonomy and control, because being able to answer the door also gives you a couple of seconds, if you know if you're on the loo or if you're in the middle of making a cup of tea that you can just, you know, finish what you're doing and go to the door. Whereas I suppose if someone could just come in, it takes away that autonomy a little bit, doesn't it?” (P5)  “The simplest solution is to carry a bottle of spray, isn't it? And just get that get that done, because you're going to be touching the door handle. And once you're in there, you're probably going to be providing personal care anyway.” (P5)  “No delays, Usually, if people are going out, then the key code would be passed to the upgrades, the upgrades or passes passes into 111. And it usually works pretty smooth for To be honest,” (P5)  “And in terms of what works well, with that current entry system? I mean, I guess it's it's, everyone kind of understands it's, you know, the key, the pin pads, everyone kind of knows how to work them. If someone's able to answer their own door. That's fairly easy, isn't it?” (P5)  “I think the good thing about the current access is that not anybody can enter the building. So obviously, if you're a staff member, and you're there day to day, then it works well. And you can go in and out as you need, obviously, because that's part of your job role. And that's what you need to be able to do to in order to do your job role. But any sort of visitor I think it's a good system to have simply because we can we can control where they go who they are, sign them in all that sort of we know who they are” (P7)  “before we go into a house, we put gloves on, okay, and a mask on a visor, and a plastic apron. Okay. And if we know the house will also put shoe covers on. So from that point of view, if we're touching any keypads there were already gloves […]we carry hand sanitizer with us. And so when we done the PP before going in, and when we're leaving, I take mine off as near to the door as I can. Not everybody does that because you know, people don't have bins outside and, or if they do, it's not been they want you to put your rubbish in. ” (P6)  “I guess the only benefit from I'm guessing their perspective is that you're not having people walking in randomly, which is obviously isn't appropriate. So it's not like I could just gain access, you know, just randomly, but then if they were expecting me, then you know, they kind of know that I'm coming and who I am and that I've got permission to be there” (P8)  “So obviously, putting a PIN code into a into a door lock is is really, you know, routine common behavior. I just wonder it will be interesting to see if it is installed in that kind of environment, whether people instinctively go up to it and you know,” (P8) |
| Benefits to FRT (36) | FRT benefit –safety for care homes and community care (5) | Benefit – thinking about spread of Covid (4),  More secure care (1) | “Because you know, we don't have to touch anything. So where there are sort of the key codes, panels, you know, people still touch, which potentially could spread COVID” (P2)  “But I think there's a whole host of stuff to be done around that, which will make the delivery of care much more secure.” (P3)  “Minimization of cross contamination.” (P4)  “from an infection control point of view. That also sounds really good, like not having to touch a pin pad. Yeah, especially I think we've all become a little bit more conscious of germs and viruses and what have you these days? Thanks, David. So yeah, for anyone going in and out, and and particularly maybe staff as well, who are going out in and out everyday and touching the pin pad? Yeah, it's a fantastic opportunity to reduce that potential for Yeah.” (P8)  “So it was one of the I was looking into the key lines of inquiry, and especially around safe. And how we can, as a common carrier as a whole could look at increasing the safety of our homes, both for our staff and residents, safety with regards to entry and exit of our buildings, access to restricted areas and the safer method and the IPC reduction.” (P1) |
|  | FRT benefit – Efficiency for care homes and community care (27) | Staff not knowing codes due to code changes, flexi staff, agency, visiting staff (8),  Family and friends ease of visit (2), Accurate staff logging (3), Saving staff time (7), Doctors ease of visit (1), Saves visitor waiting time (4), Reduce lost cards (1), Hands free access (1) | “Whereas some of the subsets are really good. They will change them every couple of months. The code that I knew was in it, me, and then they're like, No, we changed.” (P2)  “So it'd be useful for them to rather than giving them new code for each home.” (P2)  “it would benefit me in the other two ways as well, because it's not going to inconvenience me because all services I go into have that set up. What we have today is just going to allow me into the building. Because, you know, it would recognize me as a member of staff that's authorized to access that.” (P2)  “So where we have shortages of staff because of sickness annually, things like that the Flexi staff will then be able to pick up shifts in those services. So, again, they would travel around to very homes as well.” (P2)  “And you thought you go back six, seven years. Because I just thought it's easier, because the way with the up to that point that people were signing in was on a computer, quite often, if you know, you sort of walked into shift and somebody needed support, you quite often had to stop immediately, you couldn't sort of go to the computer and log in.” (P2)  “I suppose it could also benefit loved ones. visit their loved one, it would also work for them. So you know, say if there was a daughter that was concerned that their mum might have had a form or something like that they haven't got to worry about gain access into the building or anything like that.” (P2)  “And I bet you one with 1550. Staff, you wonder, we have had instances where somebody is being logged on when they've actually been home? So with facial recognition, you can't do that. […] you can swipe somebody else’s card. That’s quite a large number of people to kind of know where they are and when” (P3)  “Well, but then if you if you if, say if people arrive so if the caterers arrival, the laundry arrive, and they don't know the pin number, and you can't I hope they don't Because otherwise you got security risk. And then you've got someone who's going to let people in and having to stop what They do. And right now, that means I've got to wash their hands, sanitize themselves, open the door, let somebody in make sure they do the same. Whereas the facial recognition detection system, and then if it's the chief is that rolls up. There can be a siren going inside as you walk in the door. Chief Exactly. Again, I know, it's no never that she's good to go. It would warrant there being the additional cost, because you're saving time for staff that are having to answer the door and having to come away from residents when they could be providing care. And so the savings are going to make the cost worth it?” (P3)  “[visiting] doctors, and maybe consultants or specialists. Most of the consultants are doing something remotely now anyway. But again, that's where the facial recognition comes in.” (P3)  “And I don't know what maintenance they will need apart from the old camera clean, but I won't be every hour will it? It will be every nest is in a really dirty location. But every every every week, maybe so there's a saving in that right now as well” (P3)  “so she doesn't have to remember what these pin number either she can get in the place without too many problems.” (P3)  “Yeah, those are saving them as well. But also means it's not somebody sitting in the office waiting to go out the door” (P3)  “ So actually, when we went and asked him when we do this, we need to get into see the advantage of this. Now, actually, if you're going to say to someone else we're going to do so when you walk in the door, you also sign on. Those who are those who are abusing the system are not going to object are they because they they know they just identified themselves.” (P3)  “ link it to our day force system because at the moment we sort of, we come to the building and we punch in the code, then we've got to take our phones out, start up the app, log into day force, which can be an issue with the Wi Fi here sometimes. And then we log in. And that's how we get paid. So by coming up to the door with the camera, then opening the door and loging us on to the day force system would save us quite valuable time actually” (P4)  “I think from what I gather that, you know, for example, if one of our other homes needed for me to cover for, you know, a day next week, then all it would take would be my manager to email over my facial picture to the manager of that home, they would upload me on the system for a 24 hour period, and then I can just turn up and go in. So.” (P4)  “saving, you know, saving time, obviously, with the login into de force and blah, blah, blah, and all that having a constant record, of, you know, the current staff members within the building, at real time as well, would be good.” (P4)  “I think it's a really good idea. And it would definitely be a lot easier rather than relying on a member of staff to come let you in all the time.” (P7)  “you come into sort of a lobby, and then you've got worthy face recognition pad would be unusually on the other side of that door is somebody who works she's sort of admin receptionist. But she's not always there, because she's out about doing some pieces. So there is a doorbell which I have used before. But that some, you know, if anybody's nearby, and if they've got time to it, because they've got to come through two doors to open the door.” (P9)  “So the safety for residents, the quicker access to restricted areas for staff” (P1)  “The reduction in people losing key cards” (P1)  “Yeah, and I think it'll just be much easier, a lot more convenient. You know, when I was saying earlier about if you've got loads of stuff on you, yeah. thing down, put the number in everything up and get the door open that Yeah, it'd be, I think it would be a lot more secure and a lot more straightforward.” (P9)  “or I've got to say to you, about where we could upload, approve visitors and pre entry to appointment. Because that's one thing that I think is going to be useful is if families because at the moment you have an approved you have approved family members for visiting residents, what I would like to be able to do his family's book visiting and that their facial recognition in some of our homes where they go into a separate reception area.” (P1)  “think it would have made things smoother, much, much smoother. And I think for me, it would have made me feel less like I was an inconvenience. Like I mentioned before, we'd like ringing the doorbell repeatedly or knocking on the door. It feels quite intrusive, if you're doing that multiple times over. So me, I think that would just mean it made me feel more comfortable about entering environment. That's not mine. It's not, you know, I'm a visitor there. I'm also somebody who lives there. So I think that for me, it would definitely would have helped that and it would have just helped with timekeeping as well. Hopefully that if you could get in quicker, then you know, you could get on to next appointment quicker, and get done when you needed to get done” (P8) |
|  | FRT benefit – safety and efficiency in community care (4) | Emergency (1), Ease of access (3), End of life (1) | “They set up their own profile, it was quick to do. Paramedics can potentially do that to gain access to, like a house. So they've been called someone pulled a lifeline set off the alarm and an ambulance is gone out to home, but they can't get access because the person is living on their own they've fallen over broken their hair, if they can quickly sort of set it up, and then that would then give them access. Yes, potentially, that's, that's quite beneficial when an emergency.” (P2)  “He has a carer visit and they've got like a lockbox on the door on the wall. I mean, if they have a little camera installed, as long as obviously the weatherproof and sort of robust. Yeah then the carer would obviously, just be able to go up to the door and open without having keys hanging around. You know, although they're quite strong, it wouldn't take much for somebody to just break it open.” (P4)  “we had someone on a syringe driver, where they've got control drugs. So we would be going in once a day, and probably are, we have a specialized care team that deals with people really at the end of life and when they're not eating and drinking and they need personal care, washing, and shaving, you know, pads changing or incontinence, to prevention of pressure sores, pressure ulcers. It's a big part of our work. We do a lot of end of life care. If in a neighboring flats, we had those control drugs, and that person didn't have secure access. So that person who's obviously at the end of their life is, is in a hospital bed, unable to mobilize costs, usually, although they're not always the case. And there's usually some family member, but not always, some poor people don't have a soul in the world, and and if there's no availability in a hospice, then or in some some care homes, take people in nursing homes would take might take some money. But that's, you know, doesn't have happen often. So you usually rely on on family. And, and our teams will go in four times a day and carers and then we'll go in additionally to that, but there's still a whole raft of time, no one's going in overnight, unless they need an hour, two hours doctor, or we are going in before 8pm on a call. huge part of our community business because people want to die at home hospitals wants people to die at home. People want to be discharged from hospital to die at home with their family, ideally. And that's what we we very much do that a lot of our patients are dying at home support, people who are obviously the end of life care, they're not going to be so up to going in answering doors or unlocking doors or attending to doorbells. And like you're saying if there's not family there, or if they don't have family, or if they're not, if they're distant if they're abroad, if they if they live elsewhere in the country, then yeah, for even maybe for them, it would be useful to have, you know, some kind of security in place where they feel, you know, they know mom and dad's you know, or grandma grandpa's in an awful position. But at least they're secure. And at least people can get in medics can get in as and when they need to. Exactly.” (P6)  “Because we have key cap, we have key codes with key access, where our kids could go with one or more non mobile residents that are, you know, have no family and friends to assist in opening the door. So for me, it could be utilized for those who want to stay within their own homes, but aren't able to mobilize to go and access and open their doors” (P1)  “sometimes we need to see people every day of the year, 365 days of the year as well. So that would be great, just to be able to go get diabetics, people are often on a six month injection said prevent blood clots, things like that. So for people where it's kind of longer term, but like you're saying six months, then it could be it could be a value for those patients.” (P6) |
| FRT suggestions (10) | FRT potential additions or improvements (10) | Temperature camera (1), Integrate with other systems (7), Let visitors out also (1), Notify internal staff (1) | “build a heat camera in and take intervals on somebody's temperature.” (P3)  “ linking with the nourish system with the fire system” (P3)  “We've got this new bit of software in the company called day force, as I said, and we're not using it to half its capability that moment. Now, the project manager, the lead on that is left and we got a new one in there. And actually, that's one of the things I'll say to him when I spoke to him last week, but actually, you know, Hi, can we link those two things together?” (P3)  “If all we do is do part of it. I think there's an opportunity to look at the whole thing” (P3)  “I think I think the other thing we could use it for right now. So I think the clinical stuff. Drugs cabinet, actually. So I'm on duty today. I'm nursing in Harris Laffer and I've been among nursing in Harris and I have access to the drugs cabinet. And it sees me It says LC and Harris is the drug cabinet, I take out a set of drugs. And I then hand it to a patient who's Mrs. miggins. And there's a picture of Mrs. miggins. And we know we given that those drugs to Mrs. miggins really secure.” (P3)  “a system that knows who's in the building is going to be helpful in a fire as well, isn't it? Absolutely. What and again, we're going back to actually, Joke's on john in one way, or they got the wrong not whatever, that whatever they've done. They've gotten rid of the night issue on site. And you're now running around trying to find somebody who doesn't actually exist. Yeah. So yeah, that's an asset. Yeah, okay. We've run into problems. Every time I sit and talk about even going to be I can still think of new things” (P3)  “I think it's fantastic. It's going to make life so much easier, and especially if they can link into our we've got a day force system. I don't know if you know about that.” (P4)  “link it to our day force system because at the moment we sort of, we come to the building and we punch in the code, then we've got to take our phones out, start up the app, log into day force, which can be an issue with the Wi Fi here sometimes. And then we log in. And that's how we get paid. So by coming up to the door with the camera, then opening the door and logging us on to the day force system would save us quite valuable time actually” (P4)  “ saving, you know, saving time, obviously, with the login into de force and blah, blah, blah, and all that having a constant record, of, you know, the current staff members within the building, at real time as well, would be good.” (P4)  “Because that problem with the old, the current system that they've got now is quite often, if you don't know the PIN code, you can, I've been trapped in many, many cases where you just can't get out. Because you don't know the pin number. And quite often the doors are, you know, they are locked. So to actually get back out again, it's not just about coming in, it's actually as a visitor getting back out again. And then you have to get involved with somebody to let you out which you're then presenting more of a burden on them. So yeah, in terms of being able to get out if you can just, you know, show your face to a camera. That'd be fantastic.” (P8)  “it would be good if it can notify the person that you're trying to say. So say I was going in to see the manager or the activities coordinator that it would make it hang on their phone or something that you know, KGC she said to you for the lead group of women. And then at least they know that I was in the building, I'm just thinking quite often that that big, big places and the manager's office can be you know, down the corridor or turn left and right second door on the left or whatever. And you don't necessarily always know if even if there'll be a let alone where they are.” (P8) |
| Concerns with FRT (90) | FRT concerns – technological (25) | Changes to facial features (4), Depends on electricity (cost) (3), Power cuts (2), Internet (5), Device failures (3), Weather (5), Flashing light - epilepsy (1), Staff without smartphones (1), Night time (1) | “where people change features of their face.” (P2)  “You know, if somebody takes a selfie, and they've got sort of like, the gentleman in that video clip had quite, quite long hair with it parted. No sunglasses, no facial hair. And then if one day he cut his hair short, you know, shaved his head had sunglasses on how to full beard. Would it still recognise?” (P2)  “Say somebody decided to have a tattoo on their face” (P2)  “So I suppose the question is, you do have to regularly have your photo up you updated? And if you did make any any drastic changes to your appearance, and perhaps there should be a requirement here that you you would have to do another selfie? Yeah. Yeah.” (P2)  “I suppose the only challenge is anything technology base is dependent on electricity. Yeah.” (P2)  “And if you have a power cut, nothing works unless it's almost like a backup. So that would be that, that's the only thing that's sort of jumping out is saying there was a power cut, and I don't live that far from the [site], I'm only sort of half a mile away. And in the village, I mean, we regularly have power cuts. And they might be very short just for a minute or two. But sometimes that we have had sort of three days with electricity, because we're quite high up. And so we get quite strong winds. And it's a very old village. So a lot of power lines will sort of go down and things that would be concerned, what would happen if the power cut out? Would it be a case that all the locks unlock? Yeah. Or would it be that potentially we've got No way of actually getting in? Or are there still the keypads or some other alternative?” (P2)  “Yeah, yes is one of Wi Fi and you know, there is another issue in it of course,” (P3)  “The only thing is like, is there you know, is there a backup for if the system, you know, we get power cut, for example? Yeah. That kind of issue? I don't actually know, cuz we've never had one I don't know how the current keypad system would cope if we did have a power outage? That's the only sort of thing I can really think of on a negative aspect.” (P4)  “Yeah. And so we, again, with our solutions, we'd love to center that sound and technology. And even with when we're talking about putting SIM cards in the people, we're then relying on the mobile phone signal. And not everybody has good, as you say, everybody has WiFi. And if they do have a mobile phone, then you have to pay for data. So would there be a chart and additional charge? If people have to use data and what they want to what they want to pay for that?” (P5)  “What happened with what happens if the battery goes down? Nobody can get into my mom's house. What happens if somebody tries to steal the camera? And the doors locked? Yeah. So all those things are real concerns for people.” (P5)  “So what happens when it doesn't recognize it?” (P5)  “What if I, what if I forget my glasses one day? will it recognize my face? What if I have my hair cut? Which I need? What? What's good? What's going to happen? What if i grow a beard?” (P5)  “You can't necessarily use that in the rain, or if it's dark. So I guess all of those constraints would also be there for for their camera.” (P5)  “And people in the community, there's no control over whether you've got to visit in the in a storm or in the middle of the night.” (P5)  “One thing, when to when you see the touch white screen and it flashes those bright lights? Does it always do that? Well, thinking about strokes and epilepsy. Warnings on television warning, this flash photography. So if you're at close to knowing and it's doing that in your face, yeah. Or epileptic, then they might find you in a heap. Right?” (P6)  “I mean, is it an electrical thing? Is it like an electric doorbell? Yeah, yeah. She's power or anything? Yeah. Unless people. Some people don't have the money to put in the meter.” (P6)  “They physically don't have money [to run electric lock with wifi]. Yeah. And, and they're burning, the burning their rubbish. And, you know. […] Hardly anyone has internet” (P6)  “Yeah, so my first thought so if a few of our cameras aren't tech savvy, or they don't have a smartphone to take photos? So if that's the case, then how do you get around that? Is that sort of something that you would need us to take over to upload a photo with a smartphone?” (P7)  “And also that I'm guessing they are waterproof? Because the water? Yeah, a lot of our doors are sort of when facing so they're gonna get a lot of beating for the weather” (P7)  “I'm guessing as well, if there's a lot of rain or frost on the screen, will it still recognize the face? Or is it going to have problems?” (P7)  “If you've got a heavy frost, but it's completely because because I mean, our carers, and some visitors might have to go in the homes you know, anytime day or night so it freezes it rains a lot, especially in Cornwall and you know, I don't know if that would make it sort of not I'm not seeing a face properly if that makes sense.” (P7)  “I suppose if they're going in at night as well to have lights or I guess generally I'm not sure if they all did or not might see my if they don't then it'd be very dark, wouldn't it? Yeah, that would be something for them to consider as well because obviously your face is not going to be as recognizable with different shadows is it?” (P7)  “I was wondering, what would happen if it doesn't recognize you? How would you get in? I don't know how likely that is to happen.” (P9)  “I don't think so. I mean, is it? Is it Wi Fi operated? Or is it just how does that work? Good question, actually, yeah. If the Wi Fi is down. Don't create a problem.” (P9)  “So when we do have Wi Fi, in my experience, that are the ones that I visited, I think there was only one that turned around and said, We don't have a Wi Fi connection. But it was often quite unreliable. So for example, in the canes are quite old. So that old buildings, they got Ed, especially here, they've got thick granite walls, quite often in connection, their Wi Fi signal just doesn't stretch that far into the home. So they'd say, yeah, we really love this device. And we can get it to work in the front living room. But we can't get it to work in, you know, Mary's bedroom or the lounge at the back of the house or wherever. So yeah, I guess the Wi Fi connection would have to be strong at the front door. And then if you had it placed on internal doors within the home, you'd have to make sure that that Wi Fi is reliable as well. Because I mean, if what happens if the Wi Fi goes down? Or it's not connecting to that system, if people are getting stuck, or not able to get to somebody, then that's a really significant problem.” (P8) |
|  | FRT concern – logistics for care homes (26) | High volume of users (9), Admin required (4), System wide approach (1), Many cameras required (2), Staff learning the system (1), Price (6), People accessing without staff control (3), | “So through the course of the week, different carers and well, through the course of the day, over sort of three, four visits, different carriers will be going if they weren't sort of standalone units that didn't talk to each other. How would we program each one to make sure it recognizes every potential carer that might turn up.” (P2)  “that's the next person on the run with that sort of be easier system to sort of manage because obviously, someone's going to have to be sort of uploading all that come down to the individual user with would they have to sort of upload their own sort of photo sat on a computer in control of all that, or, or is it sort of the sort of self setup?” (P2)  “Yeah, we're all all of the departments that come under support are faced so learning development, HR, finance, property maintenance, they all go to all of the services are very much benefits.” (P2)  “I'm just gonna say, is there a way that because we've got 16 homes over Cornwall? Is there a way that like the system could talk to each other? So say, a member of staff from [one site] went to one of the services in [another town], that they will just recognize them as somebody from [the first site]? And again, let them in? Or?” (P2)  “We've got 15 150 staff so we're not a small company by any stretch of the imagination.” (P3)  “Then we will have our own external, property team, caretakers, on top of that, then we’ll have outside suppliers” (P3)  “when I say staff, I don't just mean healthcare assistants, I mean cooks and chefs and, and all those sort of people” (P3)  “But that's the main group supply food supplies, they don't always come in the front door. [One site], we have a side or back door to the kitchen” (P3)  “Probably the next largest group will be visitors” (P3)  “So I think Who else would I think oh, and then this is obviously if we get an emergency like an ambulance or, or something like that.” (P3)  “we've got some elderly carers, so it's going to take a bit of a while for them to grasp it. But you know, we've just implemented the nourish system, which is like a care, like a daily narrative of the care of the clients, which is all app based now. So you know, some of the elderly carers have really grasped that and, you know, move forward with it” (P4)  “worried about the money. I understand that too” (P3)  “It all depends on it will be very highly dependent on pricing care homes as well. And you've got to think that care homes, they run on very, very tight margins. And so thank you put the keypad on, that they know that they wipe after every entry and exit. That's cost them 25 pounds. And it's there forever. And it's protected, isn't it? Isn't? It isn't dependent on weather and everything else, then they're going to have the keypad? Yeah,” (P5)  “I guess the pin pads, they don't cost anything to maintain, do they once they're there, they're there. So it's kind of more of a one off cost […] And it’ll be cheaper than the cameras I guess” (P3)  “But of course that you know where are we going to manage on 25 megabytes now we need 125 megabytes to make it work. So we are having that it system but once you've got that in place once things like this will run off easily. So you know, is part of a bigger fortune. In that sense,” (P3)  “so they would need to be someone within within the care service or within each home that was responsible for? Yes, that's someone we're going to approve, or that someone we're not going to approve?” (P7)  “I mean, from a fundraising point of view, as I see it as a fundraiser, I would want to perhaps fundraise or get the costs funded for rather than it coming out of our pot, which is already, you know, struggling, if you like, because we're we're focusing on other aspects. products coming in or, you know, to to enhance the lives of our lovely residents” (P7)  “And I think another thing I would say is that because our carers are busy out on the floor, if you have somebody that walks into the home that's looking to visit someone at the moment, if you answered the door, you can control that person, you know, not in a bad way. But you can tell them to say please wait here, we know who you are, sign you in. If you've got somebody that literally just facial recognition and walks in and you just suddenly you've five minutes late, do you think they're you know, looking at who are they in this house, especially when you got hired as a dementia? I could potentially really upset especially they start wondering, so the only thing I would say, with facial recognition, but I guess it'd be up to our homes would be will they still answer a door to a visitor rather than have facial recognition to stop someone random walk in? Or would they really happy with that, but I think it'd be more up to the homeless, state their clients as we've got different clients with different needs. We've got some clients with very advanced dementia who are sort of, they have a lot of behaviors that maybe wouldn't be appropriate for someone to walk into the home. So they would want to answer your door still, but then you might have another home where it's very open, and clients are allowed to go in and out all the time. And that might be fine. So it really does depend on our home.” (P7)  “So it's better to have someone who leaves the building as a visitor, it doesn't really know the place or they're not staff. But they're they've come in because of facial recognition, that might not be a good idea. It might be better to actually still answer the door to them know who they are. Ask them to sit, you know, so we can we control the situation.” (P7)  “But again, the one area that we would need to be able to work on is that we established a pool of staff, because rounds can be split between people, they aren't always the same person that goes that go out. Yeah. So you would need to upload your established workforce as a whole to each of the unit. Because we work in clusters, and because the vision going forward is that care homes, don't care in the community work as a team. So you have the enry outreach, and there's fluidity between between the two, it would mean that there would that the system would need to hold a large amount of staff facial recognition data.” (P1)  “No, I mean, I, I like the idea of it. It's really good. I'm just thinking would it be? So for example, if I just thought about if I went into the [care home] or wanted to go into the kitchen, each one of those doors has a key and would be face recognition on each one of those doors, then, though, every door now that has a key code would have a face recognition?” (P9)  “I did wonder does it say when you get let in? Does it notify somebody that you've been let in? Because I can imagine, you know, maybe you use your face to get in? And then you're in and then you're kind of like, Oh, yeah, is that person then gonna come in,” (P8)  “one challenge I get, I can foresee is that we would need to establish a framework with our agency providers that they ensure that they get they gave us the required facial selfies, so that we could upload the facial recognition of the agency worker to the camera, because that could be one of the barriers that we that I foresee. And what we would need to do is make sure that there is a quick way of updating it. Yeah. Yeah. Especially the high turnover. agency staff. Yeah. Yeah. staffing. So for me, one barrier or problem that I foresee is that it needs to be reactive enough to be able to work by changing for workforce. Yeah.” (P1)  “Good question. And maybe just the process of uploading the faces, I guess, like there is some kind of human labor involved in that. And just making sure that that's as easy as possible. And whether they have to do that at the care homes as well, if that's something that they've got something that you need that they've got to approve it there. And I'm just thinking about the kind of capacity to do that. I can see that with maybe as being a sticking point for them.” (P8)  “So the care home will have to schedule times and dates of visits. So like you were saying about, you know, the back end of that, you know, there's obviously going to have to be some staff time in terms of managing the system. And I think that's important. Because it's, it's asking extra, isn't it? So it might be saving time, like you mentioned, with answering doors and whatnot, but someone is having to schedule visits on the systems” (P8) |
|  | FRT concern – logistics for community care (24) | Community logistics, many visitors (8), Price (11), Emergency (1), poor staff retention (4) | “you're out in the community, they don't go to just one person's house, they're going to be going to potentially 1012 people daily, and would each one, pick them up, but also it's not one carer allocate to that person” (P2)  “Yes. It's got to be cost effective, but it's got to be effective. So the biggest barrier for me is how many people's photographs got to be coded? It's got to be GPS, are they going to want their photographs on things? I don't think so.” (P5)  “what we were used to working with customers who have ring doorbells for example. Okay. So we got one customer where we ring the doorbell and their family sees that we've arrived. But but that thing exists, that's there already that doesn't cost very much. That's just the 80-100 pounds, whatever it is to install and then they come Security if you know who's going and who isn't because everythings recorded and gets sent straight to your mobile phone. I think the touchbyte I really like the concept was I'm not sure how practical it would be certainly in the community.(P5)  “Yeah. And so we, again, with our solutions, we'd love to center that sound and technology. And even with when we're talking about putting SIM cards in the people, we're then relying on the mobile phone signal. And not everybody has good, as you say, everybody has WiFi. And if they do have a mobile phone, then you have to pay for data. So would there be a chart and additional charge? If people have to use data and what they want to what they want to pay for that?” (P5)  “Cost will absolutely be cost a bit bit. Because you've got to think that's why do I want less than puts it? I can see why I want to put this in because I want to make money out. But why would I want you to put this into my house, if I have it? If we already have an entry point, which is a key when we can have access to a key” (P5)  “I think the NHS would also be looking at if he tried to try to sell it into the NHS. Again, I think it would be about all local authority. Why would we want to pay for you to go to someone's house, more than for a key safe?” (P5)  “There's got to be Yeah, it's got to be any any intervention, innovate innovation has got to work cost effectively against the alternative, hasn't it?” (P5)  “But it's the balancing the cost. The I guess the biggest thing is got to make it desirable. And we make it out, we make it desirable.” (P5)  “So if it was a way of entering my office at work, I may see it as gimmick. Okay. Yeah. No, that's really good. But I wouldn't pay for it because I could get a key.” (P5)  “If I was having people who, even the best of care companies, who only had four or five people who visit three or four times a day, if they were paying for it, fine. You pay for it, and you can let you come into my house. If people are asking me, who's already paying for a service, to pay more to allow people who are coming in to provide that service in? I'll tell you to get lost. And I think that's that will be an issue with the local authorities. Well, yeah. Because they they're already trying to drive cost down. Yeah. So for them to have a monthly a monthly or weekly payments on top of what they're already paying out, simply to get somebody into a house who's got the keys say, I don't think we want to go for it.” (P5)  “ put this on people's doors, so if you think about the issues we just mentioned about keys, and lots of people having keys and not knowing and some organizations having to change the carer at the last minute, you would have to upload a lot of faces to this system. Because some organizations just don't guarantee who's going to be visiting.” (P5)  “The other thing is with with touch byte, is you may well say that Hannah could only go on a Friday at three o'clock. But Hannah may be needed at 12 o'clock on a Thursday, or Hannah may ring in poorly withh half an hour to go somebody else needs to go Yeah.” (P5)  “And you have to call 111. Now, very often in domiciliary care, when people call 111. Our carers can't stay with the person, no matter how poor they are. We we try to we've never left anybody yet. But so they will say now I've got 20 minutes. And that's all I've got. So I've left the person there in the recovery position. Hopefully, they'll still be there when you get there, when you get there. And then an ambulance will be called or the GP will be called or district nurse. And it could be any member of the district nursing team. So if you got 20 members of staff in that district nursing team, any of those people could be going. So it's just this country very, very complicated. To get into work well, without having to also have a keypad or a locksmith key safe. As a backup system of key safe as the backup system. Why would you use the touchbyte system?” (P5)  “Particularly in domiciliary care, and people. It's such a transient service, that lots of organizations will have people who work for them for a month, and then disappear. Okay, right. So yeah, so if there's a cost for each person being loaded onto a system, they're not going to do it. No. That's not going to happen.” (P5)  “you're the problem with, with our, our retention is very good, but we only take on people who are already professionally trained, or we train them. But over COVID, for example, lots of organizations that have taken people in who were hairdressers or cleaners or have been, have been at throat wanted to make some more money. And now they they've left now, because they're going back to the hairdressers or the organization that the clean point, etc. So really, is that transient? Is this not a? This isn't a straightforward? I'll go and work here. And then you'll be there for two or three years. Like in most in most jobs that we have?” (P5)  “The majority of people will have other health care professionals practitioners visiting.” (P5)  “That takes someone within your service to actually approve selfies and slot them into times and slot them into houses. So that's actually it's actually a job in itself, isn't it?” (P5)  “The actual logistics of using it uploading people you're not getting you're then you might be saving a second or two at someone's door. But for a member of the team, you're you're giving them a whole extra workload, right?” (P5)  “So so if you if you've got a care agency going in, you know, poorly paid a lot of young people doing that job, because it's what they can get, you know, I meet a lot of these young people. And, you know, obviously, that they're doing a great job, but they're not going to stay in it forever. Probably. specs, printers, not much hope for them. And, in essence, quite big turnover. Yeah, absolutely. Whereas I think young people would probably be more aware of cybersecurity than older people. Yeah. Yeah. There's certainly quite a lot of turnover in care staff.” (P7)  “There's bank, bank staff that come in to just do temporary work when, you know, it can be for a day, or it can be several months to cut to cover posts. So, you know, there's a lot of influx a lot of change all the time.” (P7)  “ there would be a number Yeah, be a number of people. And as things open up more services go in. Then obviously this there's lots of outlying occasional people like podiatrists like speech and language they might only see someone once yeah But they need to go and assess the swallow I say on somebody who had a stroke, and things like that. ” (P6)  “I mean, is it an electrical thing? Is it like an electric doorbell? Yeah, yeah. She's power or anything? Yeah. Unless people. Some people don't have the money to put in the meter.” (P6)  “They physically don't have money [to run electric lock with wifi]. Yeah. And, and they're burning, the burning their rubbish. And, you know. […] Hardly anyone has internet […] no they don’t even have mobiles” (P6) |
|  | FRT concerns – perceptions and data security (12) | Attitudes towards the technology (7) Consent from those who use it (1), Privacy, attitudes, spying (2), Unsettling (2) | “I was really sort of positive around that a lot of the staff at the time didn't they were like, Oh my god, you get my DNA getting my biological data. And so they just did not understand how it works.” (P2)  “And the same way as when I experienced the hand scanner how people automatically thought that intimacy in a sample. It's just that conspiracy theory mentalities. Sinister going on. Yeah.” (P2)  “Again, is more family of the people using it is around consent. Yeah, I can I can picture a lot of people instantly, instantly settle down.” (P2)  “And when I first got this phone, because up to that point, it was the thumb. Because it's a new model, I was I was quite unsure. And I thought all what was my face on sort of on a table nearby somebody and it reads my face, unlocks, and somebody snatches my phone, runs off, but it doesn't do that. It you have to sort of properly hold it up in front of your face, it won't just sort of pick up your face and unlock.” (P2)  “Do I want to face cameras all around the house? Or in the home? Probably not. It's more important getting in and out. And on the on the doors and stuff, I think would be like, the kind of the problem with having too many cameras or too much monitoring, then people think you're spying on them.” (P3)  “Absolutely, I think that they, I mean, they're used to having zoom meetings, etc, now, but to have the big resistance of my staff, they... It's not just GDPR is how people perceive things to work as well as for them to? If so, pardon me, if you're caring for 20 customers a week, your face is going to have to work on 20 different locks per week. Wait, which? Which sounds like a nightmare to me.” (P5)  “not everybody is comfortable with with new technology. But some people are listening to it. I don't know if you were listening to with a long drive yesterday. So listening to all sorts of there was a chat, one of these talking shows on radio, too. And they were talking about older people being expected to keep up with new technology. And some people will just say, look, I just don't get it this too difficult for me. And so, there may be I'm not saying there wouldn't be but I think that some people will be very concerned that somebody can just turn up at the house and enter without having a key. How does that work that would concern them and others who would be concerned because they aren't used to that sort of technology. They want someone to ring the doorbell they want to go and answer it themselves. They want to know who's there. It may well be that for others that look that they think is a great idea. But new technologies quite, we, because we we've been using it in work now for the last 20 years is something that we come across every day. But lots of people who aren't in universities or public services don't use technology every day.” (P5)  “Absolutely, absolutely does. Is that if, if you're older and you and you can't remember what time somebody is coming, and then all of a sudden somebody appears in your house. That can be really scary to” (P5)  “And again, it's down to staff that are tech savvy, if you've got a staff member that's not comfortable with technology, they're going to be like, Oh, no, no, I don't like this. Because that's the natural reaction to do, isn't it? ” (P7)  “And I would my first concern would be an elderly person who doesn't really understand technology. And that's not all of them that's not being ageist or anything, but some of our clients don't. And they might think that's unsafe, they might think that anybody might just walk into the house with this camera” (P7)  “I don't know why that would you think of it obviously, that your fingerprint is a way that you can be uniquely identified. But there's something quite impersonal about it, it's on your hand, it kind of like I can't really see it, if I look at it, like, whatever. But this is my face. And this is how people identify me as being me. And they're looking at me. And it's just quite Yeah, there's something there. You've heard, like very personal about your face. To say that in any other way. Yeah.” (P8)  “I was when I was watching that video, it was having some thoughts like, Oh, I wonder if I upload my face? And then suddenly, I'm on a brochure for a care home?” (P8)  “Yeah, I would, yeah, maybe I would want a little bit of reassurance about around data protection. And so when I uploaded it, it would have some kind of thing like, your face won't be used in marketing materials for you know, this, that and the other, also marketing materials for the actual product itself. So for touch by Oh, yeah, I wouldn't really be very interested in having my face plastered all over their marketing materials, I don't think or being used for any other purpose. That could be slightly more dubious, I suppose. So yeah, I'd be looking for some kind of assurance that my face wasn't going to be used for any other purposes.” (P8) |
|  | FRT concern – safety (3) | Emergency (3) | “It was automatically in sort of emergency let you in. But my only concern with it. […] Or there could be a system where I don't know if like, say, paramedics, their ID or something like that, then it would read their ID cards. So it's like an emergency feature that they set, put something up to the scanner. So rather than actually having to recognize their face something generic that they would get issued as part of their ID or that could also be read same to the police fire. Emergency services that immediately gives them access.” (P2)  “So I think Who else would I think oh, and then this is obviously if we get an emergency like an ambulance or, or something like that. […]the ambulance drivers and paramedics in Cornwall get a special card that they wave in front of the camera and lets them in” (P3)  “So if you have an emergency situation like a fire or an ambulance definitely needs to come in. Then how does that work to say would that recognize an emergency service bystander? And go? Okay, let them in? That sort of question. Maybe I'll have as well, the ambulance residents and staff?” (P7) |
| FRT Acceptability | Current FRT/biometrics acceptability (4) | FRT on phone (2), Previous experience biometrics (2), | “my phone uses facial recognition to unlock. And I find that so much easier. ” (P2)  “So I have no issue with sort of biometric data, my phone uses facial recognition to unlock.” (P2)  “in a previous job, I've had experience of biometrics, but it was, it was a hand scanner. And it wasn't used to access buildings, it was used to sort of clock in and clock out and start your shift.” (P2)  “Honestly, I'm a real advocate of the facial recognition. I think that is absolutely fantastic. Time to move with the times” (P1)  “No, I mean, I've got an ID card. So my photo is already stored somewhere. So you know, it's everywhere. It's pretty hot on GDPR. I haven't got any concerns about that wouldn't be.” (P9)  “So I have no issue with sort of biometric data,” (P2) |
|  | Personal acceptability to proposed FRT | Would use system at work?   Would upload own face? | P1: Yeah  P2: Yeah  P4: Without a doubt  P6: It would be useful just to be able to get in and give him the help that he needs  P7: I think it's great. It's great. I mean, it's great not to have to touch something, and it would be a lot easier for someone like myself that could just go brilliant. I could just go to there.  P9: Yeah, definitely  P1: Yeah  P2: Yeah  P4: 100%  P6: I certainly would be happy to have face ID.  P7: Yeah for sure  P9: Yeah |

**Table 2: Full table of evidence from post-implementation interviews**

| **Theme** | **Grouped codes** | **Initial codes** | **Example Evidence** |
| --- | --- | --- | --- |
| Benefits of FRT for residential care settings (53) | Improved customer or resident autonomy (7) | Accessible without staff, autonomy (6), well adopted by residents (1) | “I just think it's nice, it's a nicer experience for them. Our customers, isn't it where, you know, I think sometimes they don't necessarily need to have staff presence to go and open a door. And they can just go straight up to where they need to go. And it gives them more independence.” (P13)  “No, it is because our gentlemen can come and go alone, because at the moment, nobody's got the key to the outer door. So it's a benefit for the customer ease. You know, they don't need us to open the door for them yet. Even though there's somebody here 24 hours a day. There's always a member of staff here. But for them, it's helps with their independence” (P10)  “Yeah, I know. One for [Customer] […] he gets a bit bored. He goes outside to check his post box 10 times a day. So yeah, so he's, he's loving the independence of being able to go and come back and not bother us as such” (P11)  “[Customer] loves to rock up sometimes, like midnight. And, you know, we don't have to answer the door. So it's, it's given them more independent. Yes. You know, it is really structured towards the customers as well. Isn't that. Yeah. Which I love. So we are independent living service or supported living service. So it just gives them the freedom and it's not like, ‘oh miss I’m going out’ or ‘miss, can you let me out?’ Yeah. Because, yeah, so yeah, it just yeah, it's really good for them. It's a proper, it's like a block of flats instead.” (P13)  “Yeah, cuz it's, it just gives them their independence, didn't it? They? They don't have to knock the doors going out. Or can you let me in? Or it? Yeah, it just gives them. And it probably gives them a bit peace of mind that they're not bugging us all the time? Because they apologize all the time. They say sorry. I say, you stop. No, no problem. Yeah,” (P13)  “Just add into that as well, when we look at future commissioning services. So the long term plan here is, eventually we'd like to rent out this flat. So and we'd be a little bit more remote. Um, we're not there yet. One, because we need to find somewhere close enough that we can respond to if we need to. But I certainly think for the future of this type of supported living schemes and, and similar. I like the idea that again, it again, it gives people a bit more autonomy” (P12) |
|  | Improved efficiency for care settings (23) | Saving staff time (8), avoiding delays waiting at door and faster access for staff and customers (6), financial savings (2), too many codes to remember (3), hands free (2), cost (1), responsive to staffing changes (1) | “Because sometimes I mean, we do have a gentleman at the moment, two to one. So when he's in the vicinity has to be two staff, because you cannot just go and answer the door and leave him with one person say, yeah, there probably is a delay, you know, and it is frustrating cuz we're all watching the clock.” (P12)  “I was gonna say it’s cost effective, and with time management is cost effective. It frees up more time for us to be doing the workload that we have, rather than Like I said earlier, I often answer the door, sometimes, like you answer, then you answer. And it's always a constant flow. So with the face recognition in place, it just gives us a lot more freedom to, to continue working.” (P13)  “So like for us because sometimes it might only be myself in the service manager here. And we're really busy. So for us to keep getting up and opening the door, you know, especially like if we're in the meeting. So to have the face recognition for people to let themselves in, helps us” (P10)  “I think if I, if you if we have one of our housing colleagues here, they would say that it's probably saved on the cost of replacement of locks and keys. Okay, so it's not unusual for people to lose their keys. It's probably one of the biggest expenses, right you can have. So just by taking away the fact that they don't necessarily have to take care of a door key, obviously, they need them for their own flat, but it's a bit more manageable. You know, we'd have to be replacing a front door, and their flat. Yes. At that stage, so I think definitely saving our housing colleagues.” (P12)  “So the benefit is that I can get in and out of the main door into the building really easy. So in the building alone where we have key code, locks, we've got about six different codes. And even though I've been here now, this is my third week that I've been based here, I don't know all of those key codes. There's some areas that I still can't access freely without support from staff. But it's nice that I can come in and come out of the building without having to sort of call for staff or wait or anything like that. I don't have to, you've not got to remember or learn when it gets changed and things like that.” (P2)  “It's just made it easier really sort of getting in and out if you don't know the codes” (P2)  “Getting in faster. Oh, yeah. Well, yeah, I mean, you know, within five seconds, the light goes green. Okay” (P11)  “Because I happen to not put a key in and just look at something and bang is open.” (P10)  “For example, I've picked up a couple of deliveries from out in the reception corridor there. That was beneficial, because I literally just walked up and it scanned my face. And then I entered the building.” (P4)  “If you are busy and there's a lot going on, and there are people at the door sometimes can that mean like delay waiting, you know, it's very sad” (P13)  “Because sometimes in here, it gets really quite busy. And when I'm in meetings, and all we're all in the meeting, up to the door, opens the door and then it's like, we were a new mascot we don't allow. So it's, it's, it's the constant traffic back and forth, back and forth.” (P13)  “Well, it takes a good 10 seconds by the time they ring and the doorbell goes off, right? And then like, if, if it's only me here, I know, I'm on the phone. You know, it could be another two minutes before I put the phone down. Or like, you know, if I'm at the computer, and I have to save something, you know, they're on the doorstep waiting. So it could be anything between 2,3,4 minutes, maybe? So has made a difference, isn't it?” (P13)  “Yeah, yeah, it wouldn't sort of take away the need for staff, but what it would do is free up staff, so they could then focus on other other sort of duties, and things like that.” (P2)  “Well, it's my getting in and out the door a lot quicker. [Before] when I can't remember the codes about 20 minutes” (P9)  “If anything, it makes life a lot quicker and easier.” (P9)  “And it has lower cost implementations because otherwise, you have to get your system programmers in to reprogram every single one of the keypads in the building. So it's more reactive with regards to security in that sense.” (P9)  “And then within 24 hours, myself and the other manager were added to the system. So they were very quick and responsive with regards to adding and removing f7, which is good.” (P9)  “A few things really. I think mainly for me, it's the not touching things that that you just literally just put your face in front of it. The other thing was, generally, I know I have an awful lot of people going in and out of any of the homes, you got your hands full, you literally just stand in front of it and open the door. Obviously, you can just get the door open. You don't have the things down. Yeah, in a different way. So yeah, I found that really convenient.” (P1)  “Yeah, it just, it just lets you through really quickly, doesn't it? And then it just unlocked the door.” (P1)  “Yeah. And you know, it would it would save people having to stop what they're doing to let me” (P1) |
|  | Improved security (10) | No code sharing, access only for those approved (5), no code guessing, safer for residents (2), quicker access in dangerous situations (1), staff turnover (1), different access to different areas for different team members (1) | “Some of our customers may be prone to be picked on. And even if someone is following them, we know [with the FRT], that person can get in the door. Which is nice. Because no matter how much we try and protect, outside, there's always going to be something okay, but it's nice to have once they're in the door. Nobody can get in that door, if they've also been following them. I think that's one of the nicest things for me. So I think it's a factor of safety for them. Because there will always be someone that wants to pick on somebody. And it's just the fact they get in even if they're being followed, they get in the door quickly” (P14)  “We use our display to unlock the door, which I don't like, because where our cameras turn, most of the time, you can't see who’s at the door. So you're actually opening the door for somebody who you don't know who they are.” (P11)  “Yes, I do. I think it does. It definitely. Yes, stops kind of people coming in that me like, kind of like me, that would know the code, but don't necessarily work there all the time.” (P8)  “It gives a security of a building. So you know, nobody can just walk in only people that are allowed in can get, it doesn't stop people from opening the door. Of course it doesn't, but it does minimize the risk. So I think certainly for the future commissioning, it's, it's a great idea. And I think it's something that our commissioners are gonna like” (P12)  “It kind of gives us a little bit of peace of mind as well. So we ought to remember carers, so we automatically we can't help it, we automatically have that want to protect everybody. And I think it wouldn't surprise me if you know, the team, when we do get to that point of leaving, they're all going to have some some fears of what's going to happen, how are they going to keep safe? And for me, this, this face recognition gives me a little bit of satisfaction that people are going to be slightly more safer” (P12)  “it's the element around security for our residents, because that home in particular, as residents that are very mobile and driven to walk also around” (P9)  “security with staff that change their careers. So as staff leave, it makes the system more responsive.” (P9)  “Yeah, yeah. You've got the caretakers, maintenance people, too. They've got certain areas that they use. And they've always got their hands full. Yeah. So you know, entry points, like that might be beneficial. But yeah, if it was just sort of specific to those people, those staff” (P1) |
|  | Improved safety (13) | Accurate log of who entered building (4), better GDPR than manual log (1), less infection control touchpoints (8) | “But then it become more accurate. Yeah. Exactly. Who's in the building?” (P10)  “Yeah. And again, if we if we come back, so that was community and and not just not just mental health customers, but also a learning disability. But then I am thinking as well for mental health workers was if we could, almost not not so that the CCTV, yeah, but almost so that it could just monitor people that are going in and out. So if we ever do have an issue, or somebody raises an alert, then we have something to go back? and have a look to see. Yeah, this person did come in at this time.” (P11)  “Physically input that code, you know, with the hand, which would then be an infection control risk. So as long as you're on the system, so you know, if you're an authorized member of staff and your photo has been uploaded, you just get instant access when it sort of recognizes your face. And then you don't have to touch anything, which is great.” (P2)  “And by not having to touch anything. Yeah. The thing that has changed is the infection control at that high risk area” (P2)  “You've got the infection control on the front door as well because people aren't having to touch, you know, a key code. So because it's completely hands free, you've just got a sort of wave. And for from an infection control point of view, that's probably one of the high risk areas because staff who are coming in once they are in the building, they are wearing PPE they're washing their hands, they got access to sort of sanitizer, but at the front door there, we have external visitors, professionals, you know, you're all sort of coming. So it stops that high risk area, with lots of people sort of touching. I think I think it goes a long way towards infection control, which, you know, in current climate is even more important.” (P2)  “I think the other benefit that I could really see is from an infection control point of view, because it's unlocking the door with the, you know, without having to touch a key or touch a, you know, like a key code pad. So that's gonna be a significant benefit.” (P8)  “Yeah, it's just it's just not you know, in COVID times, it's just nice not to have to touch anything” (P8)  “Yeah. I'm just thinking of it as an additional benefit. And it is COVID related. So actually, we had the fingerprint entry. We never got working. So having the face entry come in and have it working, but actually also taking away the need to touch” (P13)  “Not really, I mean, obviously, the you're not touching anything with the keypad buttons. So that still obviously is going to be a benefit.” (P4)  “complying with GDPR with regards to logging staff in and out of the building, as well as any professionals and visitors as well. So that's good.” (P9)  “That's That's it. So you know, it's good because it reduces touchpoints. And you know that I'm a great happy friend of reducing touchpoints,” (P9)  “There has also meant that we're changing if you're in and out doing lft or PCR testing, because we test in the in the corridor. So with regards to the testing it it does does help because you're obviously not having to clean the pads and everything as much as you normally do.” (P9)  “You're just going in without that sort of worry on infection control, isn't it? It's so important. Anyway, but right now,” (P1) |
| Positive reactions (27) | Reliable (7) | Reliability (3), works with mask on (1), manageable system, intuitive light system (4), worked straight away | “Yeah, no. I mean, I'm I'm in here virtually seven days a week. So I used to live next door until Saturday. So even on my days off, I used to pop in for a cup of coffee. So yes. So since it's been there, I think I've been in here virtually every day. Yeah. And I think if I remember right, is only once that it went red and then I waved, and it went green straight away.” (P13)  “It's gone green. Yeah. It's never been bad for me. To be fair, I haven't been here that long. A few months. It's never gone red.” (P10)  “That was really good in the dark as well, cuz obviously it goes green in the dark. So when it's night time, I feel like, yeah, it's still visual for the guys and the staff. Yeah, I was coming in like midnight. So it's pitch black. They've [customer] been to the pub and had one too many. It's still that green visual. I mean, I'm safe. And it still works in the dark” (P12)  “Green means go.” (P10)  “So the white lights that sort of constantly on when you wave your hand that starts to flash. And then it will turn red or green, whether it's recognized you or not. It's it's self explanatory, and is a traffic light system. Means green is go. And I think even if I was say, English wasn't my first language, again, is universal colouring. So straight away. I would I realize Green means Okay, and red means it's not recognize me” (P2)  “Yeah, yeah. turns green to open the door. Yeah. Like [name] said, green for Go Red for stop.” (P13)  “Having the colour system rather than a push button to get in or ring the doorbell. It's better for some of our guys with autism. Like I said, the schizophrenia and paranoid. They know, if they come to it and it's green, green means go. You're in. Yeah. You know, it's that simple. It is, it just works for this customer base.” (P14)  “It does work with my face mask up, which is a bonus.” (P4) |
|  | Widespread potential (13) | More widespread potential (7), use in domiciliary care (3), use for more independence (1), use for different doors to allow access to different people (2) | “I certainly think for the future of this type of supported living schemes and, and similar. I like the idea that again, it again, it gives people a bit more autonomy, it gives a security of a building. So you know, nobody can just walk in only people that are allowed in can get in.” (P12)  “I've seen carers unlock a key safe and leave the key safe open, they take the key with them into the house, but anybody walking by that, you know, you just go go look at the co-ordinates, and then you know, what it is to unlock. So, you know, if, say, I was an opportunist thief, I could then sort of go and get the code. And then at any point that I want to enter that building, I've got access to the key. Whereas something like the Touchbyte system, you know, we could load the photos up or any family members, any professionals that might sort of be visiting so like community nurses, district nurses, things like that”(P8)  “I think it'd be absolutely brilliant. And I could see it being quite widely used in in care homes, and in domiciliary care as well. I think there'd be quite a strong application for there in people's homes, where maybe they do have like a lockbox or something like that. It could definitely improve security in those situations” (P8)  “I could see it being really useful. I mean, I imagined in the case, they've got kind of like meds, cupboards or rooms, or they've got staff rooms. So you could, as long as you can upload faces to specific, you know, rooms, that only people that can access meds can go into that room, I can see that there could be some real value there. And if there's rooms that they don't want residents to go into, then that would also be really good that, you know, any staff can get in the staff room” (P8)  “Health professionals that are that are regular and start pushing it out towards them. I'd like I'd like that to happen” (P12)  “So if we had them on things like the manager's office duty room for the HA threes medication room, that would be really beneficial if you could have it that different points of entry. Getting the different locks and the different points of entry you could allocate, who was authorized to go through that door, if they were all on the same system, so that anybody who can come through the front door into the building can then access all these other areas that, potentially wouldn't work out how I'm thinking, because that medication room, you would only want the HA threes, the med trained people to have access. So it'd be good if we can have multiple ones throughout the building, but depending on what the area is that it's accessing, only certain people that are authorized to access those rooms are then recognized by the Touch Byte on that that sort of point of entry. If that makes sense.” (P2)  “I think, yeah, I feel I could use it. Like for example, I'm, I'm in the caretakers office, which is full of screwdrivers and drills, etc. So obviously, that has to have a keypad system. I think it'd be beneficial for places like the management office or caretakers offices, the laundry room, the kitchen doors, I think, yeah, if they get it, right, it's got a massive potential to to make life a lot easier around the inside of the home, not just the actually the exit of the home.” (P4)  “I think I think for me, it's it's more if this was rolled out, I think I think that would be really good. Yeah. Yeah, I would say it should be more widely used” (P1) |
|  | Adoption (7) | Would recommend (1), well adopted (2), would continue using (4), high usage, positive family opinion (1) | “I would say, all the staff that work here, are all aware of it, they're all using it.” (P2)  “I've got no negativity. I mean, so demo was positive for us” (P10)  “Our gentlemen have got a hold of it brilliantly” (P11)  “Customers love it.” (P13)  “I would continue to use it, but I would hope that the actual recognition would be able to be improved and improvements. But from my point of view, yeah, I definitely would use it is is it still easier than having to ring a bell and gaining access in that way” (P8)  “Yes I’ll use it” (P2)  “Yes, I would. But I think it needs improvement. I was really passionate about it and i still am but. Obviously, you need negative and positive feedback to be able to change it and stuff.” (P4)  “Yeah, I went to answer the door a couple of weeks ago, and a couple of the relatives were visiting one of their mother, and they said, Oh, what's that on the door? And I said, Oh, it's facial recognition, entry and exit system. And they said oh that's really good. I said, Yes, fantastic. You know, we're sort of in the trial stage at the minute and it needs tweaking, etc, etc. I said, but in the long run, you know, it's going to be a really good thing. And they were really positive about it. They were like, it's nice to see things move in into more modern times, you know, just to try new things and see if we can make life easier, even if it is just going in and out of the door.” (P4)  “I would like to see it being quite widely used in in care homes, and in domiciliary care as well. I think there'd be quite a strong application for there in people's homes, where maybe they do have like a lockbox or something like that. It could definitely improve security in those situations. Yeah, it's just it's just not you know, in COVID times, it's just nice not to have to touch anything.” (P2)  “I think the other benefit that I could really see is from an infection control point of view, because it's unlocking the door with the, you know, without having to touch a key or touch a, you know, like a key code pad. So that's gonna be a significant benefit.” (P8) |
| Barriers encountered (43) | Perceived concerns (11) | Power cuts (1), robustness (4), trust in GDPR (2), no infection control benefit (1), no guidance on where to go once in (1), logistics of uploading staff long-term (1), cost (1) | “We had a couple of issues I don't know if you're aware a little old lady come down. Off the Wall. Yeah, she come down and like ripped off the wall. Yeah. Yeah. She doesn't live here. No, no, no, no, no, she’s got dementia.” (P12)  “It would easily break, I mean, for a little old lady to tear it off the wall. You know, it did break quite easily. Luckily enough, there was wires and she screamed, otherwise it could have dropped to the floor and smash. So yeah, that was a bit of a concern” (P11)  “I think I think it could be a barrier for some people. I think people are a bit more aware about kind of GDPR and hacking, and, you know, where's my data going? I think people are more aware of that these days. So yeah, could be could potentially be a barrier for some people.” (P8)  “Yeah, we've had a couple of issues where it's broken wasn't robust enough so I know they've been out quite a few times just to take you knew imagery of it so they can go back and do a 3d block for it so it needed to be more robust, we had a young lady, or no an elderly lady come knocking yeah, middle of the night and she managed to break it so he said you know if somebody have a more mature age breaks it, our guys if they're in a bit of a tizz, they're gonna Yeah” (P13)  “I did also worry that we help people with depression. Now we have people who have mental health support needs here. There are people that have paranoid thoughts. And I I did wonder whether there would be some people here that would say, absolutely not, I don't I don't want my photograph being taken or I don't want it stored in a system or anything. I'm sure we will, we will most likely come across that.” (P12)  “My only concern is something that I sort of thought about the other day, was if there was a power cut, would it unlock, you know, like, like fire doors would? Where they just all instantly unlock? Or would it remain locked? Because then potentially, that that would be a concern, you know, in the event of a power cut, how would we then get through in an emergency or anything like that?” (P2)  “And I gave them my photo. But yeah, I still worry a little bit like, where that goes. And like, you know, is my face only accessed at a specific time, which I think it is. So in that respect, yeah, I think the security's improved, I would just want to make sure that my face was only like, active at the time. So I can go back to that at like six o'clock in the evening, again, using my face.” (P8)  “It might add to workload to be uploading and removing and scheduling visitors long-term” (P9)  “So we have a set area where they go and they don't come through the building, and they sort of walk outside and enter the sort of visiting room from the outside of the building. So that's why you know, because we don't want people just freely walking into the building our infection control measures going out the window.” (P2)  “It might be a bit of a nightmare, keeping track of them, whether they're still working for the coming to me or no, you know, whether, when we take them off,” (P10)  “ Because people are different height. You know, I know [team member] initially he had to find a bracket to put on it because the sun was glaring. But yeah, I think that's probably why it's broken a few times because people have tried to adjust it to fit their position.” (P13)  “Yeah, yeah, that's the thing, isn't it? It's the cost. Because not only is it the whole system, but it's got to be fitted. And yes, yeah, there's probably quite big costs that.” (P1) |
|  | Negative reactions (13) | Did not match expectations (1), frustrations (2), not quick enough (5), slower than touchpad (5), | “I can see there being a benefit. It just needs I think tweaking slightly to make it more user friendly and quicker. That's that's the main issue, really, I mean, because the girls, they sort of come in, they're on a mission, they want to get in, get started, get changed, get all scrubbed up and stuff. So they'll go up to the facial recognition camera, then they've got a wave, and then they're trying to get their face and it won't, it will flash red. So half the time, 90% of the time they give up and just press the keypad because they're so used to doing that as well.” (P4)  “I think it's going to make things a lot slower for people, just because it isn't, doesn't seem to be picking up the face straightaway. So you've got to try and multiple, multiple times, and you kind of can't help but move because you think that you've done something wrong. So then you keep moving. And then you're like, well, it's still not working. So yeah, you're just kind of not sure what to do to make it work. But yeah, 100% makes things Slower, slower. Yeah.” (P8)  “I think it just needs to be, it doesn't need to be quicker at recognizing your face, it needs to do it straightaway, rather than after five or six attempts. And I think that's going to be key for staff as well, you know, they're busy rushing around. I'm a visitor and yeah, I'm on a timescale. But for them, it's even more important to be able to get through in in a timely manner. And I think as well, like, if, if that was improved, then you probably would negate the need for training as well. Because if you think about every single person that comes into the home on an a kind of ad hoc basis, you probably not gonna be able to train them in how to use it. It probably actually does need to be able to find out no without training.” (P8)  “I mean, with all new technology, there's always going to be sort of hiccups and glitches and things like that. There are a few things I've noticed about the system. It's like, sometimes you have to wave your hand in front of it, to get it to activate and then it reads your face and let's you in. And I have noticed a lot of the carers are too impatient. They prefer to just quickly tap the keypad because it's quicker than using the face recognition system.” (P4)  “They're creatures of habit. If it was quicker. And they would use it more if you know what I mean, but sometimes they walk up to it and it's not flashing, so it's not reading and then they just go straight to the other side and tap, tap, tap, and then they're in.” (P4)  “Because obviously, it's not as receptive you know, really, ideally, you want to walk in and walk straight up to the camera it'll flash flash flash, unlock, then that would be quicker than going up to the keypad and enter in in six, five digits. I think that's why a lot of the carers don't use it, it's definitely not not quicker than the old button system.” (P4) |
|  | Problems encountered (19) | Camera not at right height (2), sun glare issue (4), issues in positioning face, customer lack of understanding, requirement for training, positioning advice needed (4), didn’t work (6), hand waving (1), Removing mask (2) | “Two of our gentlemen have got a hold of it brilliantly. But we have got one that we support that's struggling with that. And I think what he's trying to do is he's putting his face right up to it. And I think is getting too close. I think we need to go out there and teach him how to use it properly.” (P14)  “A lot of the carers They're very old school stuck in their way slightly. I've said to some of them you getting on? And they said, Well, I tried it a couple of times didn't work. So I just use the keypad.” (P4)  “I mean, obviously, they've gone up to it. I think in the first few sort of days and weeks because of the glitches, it might have put quite a lot of the cares off because they can be quite impatient.” (P4)  “What I was doing was kind of the locks positioned quite low. So I kind of felt like I had to bend down a little bit for it to see my face. But I'm not really sure whether that worked or whether that was the right thing to do. But then I was also moving away from it and moving forward to it as well.” (P8)  “Yeah, you've got you've got to stand about that far away. And I think with the customer is like that [close]. And then he stands by and, yeah, I think it's just too close, we're gonna have to teach him how to do it.” (P10)  “And he said, on those stand a bit further back from it, and it will, it will work better and yeah, but yeah some of the carers are literally going up within two inches of the camera. And I said that you don't have to do that. You just have to stand back a bit. And it will read your whole face, as opposed to You're so close to can't read your whole face. But yeah, I don't know. If they tweak it and adjust it, it might need somebody to sort of come out or somebody within the home to be like I didn't know the camera champion so that they can guide everyone on how to use it correctly, as opposed to going straight up to it within a couple of inches, because there's no way it's going to reach our face that close.” (P4)  “It's just the speed. And like I said, it doesn't recognize everybody's face for some reason. I've been banging onto my kitchen team, all you got to do, it's really good. You know, come on. Yeah. And every time we've come out there, it's like, no it won't work, no, it wont work.” (P4)  “And the other thing, as well, is that I have just sort of squat slightly, because of where it is. It's not at sort of the eye or face level its lower. And if I was just sort of stand up and stand back, it doesn't seem to I sort of have to come down to my faces at the same level.” (P2)  “Because the sun comes up there and goes down there. So with the sun coming beating down, we, was thinking it might be something to do with the sun in the background.” (P11)  “Think if there's a bit of glare from the sun, maybe that might affect it.” (P4)  “Potentially the sun is affecting it might be, that might be why it doesn't necessarily pick up your face clearly sort of first time if the sun's sort of hitting it at the wrong angle.” (P2)  “but where I sort of potentially think that the sunlight might be affecting how it sort of picks up people's faces, it might be that the sort of the outer casing, like the green sort of plastic that maybe have at the top, it sort of comes out slightly further. So it's almost like like a little awning or like a little prochway, which would then shield some sunlight, potentially. So it can then sort of register your face easier. But other than that, no, I think the actual way it works is brilliant.” (P2)  “We've sent over numerous numerous photographs, but nothing ever works. Right? So he's out there doing this and it's never, ever work.” (P13)  “Yeah, that's, that's the main thing really, is this the time you need when you walk up. And I don't think really, you should have to wave your hand to get it to function, I think it you should have detect you like a motion sensor. So as soon as you walk up, it knows there's someone there, which it must do, because sometimes, sometimes I've walked up because it's like, basically a white light when it reads your face. And if it does read you face it goes green. If it doesn't really face it goes red. Sometimes I'd walk up and it's the white light, and it will start flashing. But, I'd say nine times out of 10. You have to wave your hand in front of it.” (P4)  “Yeah. We did find in the early stages, that sometimes there was no life in the cameras, I mean, literally have to turn a switch off and then back on again to get it to.” (P4)  “be very careful about where they look to put the lock. Because obviously, if it's on if it's within an internal area, which is resident within resident areas, then they are removing their mask in a resident area and breaching CQ c regulations if that's within a two meter. So that does make this such a large impact.” (P9)  “So when I initially went to the green, it wasn't working. And so the face recognition wasn't working either for myself or any of the other staff. And it did unfortunately need to be recalibrated again after a couple of days, and it wasn't working for about a week. But when it was back up and working, it worked very efficiently, and I have no problems or issues.” (P9)  “But with that with the masks, and then it not being able to recognize your face but the mask on causes concern for me because the mask should be worn in around those areas.” (P9) |
| Suggested improvements (18) | Accessibility considerations (8) | Audio feedback (2), positioning feedback (1), screen to display your positioning (1), camera moves to find your face (1), not understanding lights (1), Hearing impairment and colour blindness (2) | “Maybe some audio feedback. So like I said, obviously, this is probably going to work for hearing unable to people, but yes, and kind of, I don't know. Yeah, audio feedback, like, move, even if it could say like, move forward, or step back, or unlocked, or something like that. I think that would be quite helpful” (P8)  “So if it had a screen where you can see your face on it, because for me, that looks like it's too low. Yeah. It doesn't, yes, if you could see that it was it was seeing on like a feedback screen. And like, you know, I don't know, some of the face facial recognition things you can, you've got to put it within a frame, like on the screen. So at least you could line yourself up with it. Straight away” (P8)  “I think they would probably guess that if it goes green, that means they can try the handle. But again, yeah, I don't think a lot of them stand what the different colour lights and flashing lights mean.” (P4)  “But one of the thoughts that I had was, if you and you can kind of almost hear the door unlock as well, which is quite helpful. But also, if you've got a hearing impairment, you're not going to hear that. So you're gonna be relying very much on the lights. And I also wanted, how that light system worked if you're colourblind, because it's probably going to all look white or gray, possibly, I'm not sure. But obviously with the green and red. They're next to each other on the colour spectrum. Obviously, it's intuitive from our point of view, you see colour, but if you do, yeah, I'm not quite sure if you'd be permanently frustrated if the door wasn't unlucky. And you kind of couldn't see why.” (P8)  “Could it move to find your face” (P12) |
|  | Integration with other systems (10) | Visitor log-book (1), staff clock-on system (3), fire system (2), healthcare visitors, exit camera (1), vaccination status (1), car reg for emergencies (1), Track and Trace (1) | “Eventually, in the future, it could link to [staff clock-on app] that would get the carers massively on side, because at the moment, they come in, they punch in the keypad, they open the door, they get their mobile phone now they log in to the app and they, punch in well if you said to the carers right. Okay, all you've got to do now is walk up to the camera, it will let you in and it punches you into the clock-on system that would get a lot of them on side a lot quicker.” (P2)  “And linking to the day force system would be massively, massively beneficial. If Eventually, in the future, it could link to that that would get the carer's massively on the side, because at the moment, they come in, they punch in the keypad, they open the door, they get their mobile phone now they log in to day force and they, punch in well if you said to the carers right. Okay, all you've got to do now is walk up to the camera, it will let you in and it punches you into the day force system that would get a lot of them on site a lot quicker. Because it saves them having to get their phone out of their pocket. And it's all done dusted. They're in the building. And they're clocked in. And they can crack on then” (P4)  “Who's in the building? So then that I guess, would be a camera all the way out?” (P11)  “We also were saying about the fire register, because obviously, getting people signed in is quite difficult because the families don't. Okay. So if we had to face recognition, where it would recognize family members, then that would be good for us for fire registers as well.” (P10)  “And I don't know if it's got that function or not. But I think I quite like if we could clock timings of people in a little bit going on the back of what Paula was saying, in terms of fire safety, that would be really good. And we were able to access it remotely. And that would be good. And certainly, yeah, clocking people in and out, so that we can check the time to visit something.” (P12)  “And what I would like you to be able to do is with the changes around the government guidelines, is when we set up a professional or a staff account that we as as a care provider and as for example, as a manager of that home site, utilize your system as our due diligence around COVID vaccination status, say that only those with active use have confirmed vaccination status.” (P9)  “Yeah. I'm just thinking, though, how quick would that be to call up those details. So for example, ages ago, I parked and an ambulance had to come into the home, and I hadn't realized that they could come in, but they couldn't reverse out. So somebody had to see whose car it was more find that person and get them to move their car. I'm just wondering how quick that would be to get all that information up without opening the book and going like that they're blocked car,” (P1)  “Track and Trace. That is actually. That would be really good.” (P1) |
