# Supplementary material for: Facial recognition lock technology for social care settings: A qualitative evaluation of implementation of facial recognition locks at two residential care sites
Source: Front Digit Health. 2023 Mar 3;5:1066327. doi: 10.3389/fdgth.2023.1066327 (PMC10020502; doi:10.3389/fdgth.2023.1066327)
Supplement: Supplementary file 2 [file Table2.docx]

**Appendix A - Facial recognition lock technology for social care settings: A qualitative evaluation of implementation of facial recognition locks at two residential care sites**

**Appendices:**

**A1: Pre and Post implementation semi-structured interview schedules**

**A2: Prompts for process mapping**

**Table A3: Displaying content analysis and example evidence from pre-implementation interviews**

**Table A4: Displaying content analysis and example evidence from post-implementation interviews**

**--------------------------------------------------------------------------------------------------------------------------------------**

**A1: Pre and Post implementation semi-structured interview schedules**

**Pre-Implementation Interview**

*Interview schedule:*

Confirm the consent form has been signed, happy to be recorded, **start recording.**

Introductions, name etc. Semi-structured prompts:

1. Participant role (e.g. visiting nurse, hairdresser, friend, family, staff)
2. Please tell me about your current experience of gaining entry to the care home? (e.g. do you ring a bell, use a key fob, staff card).
3. What works well with the current entry system?
4. Are there any challenges with the current entry system? Do you have any stories of challenges you’ve encountered? (prompts; costs, waiting times, risks of medical delays, frustrations, infection control concerns with covid, norovirus and flu, fewer contact points, reduced safety of lock boxes and pinpads, issues with cleaning pin pads?)
5. I’m going to show you a video on a proposed alternative, the company behind the innovation are Touchbyte and they have developed a facial recognition lock that may be useful in care settings (**play video**), what are your initial thoughts on the facial recognition locks?
6. Do any questions spring to mind?
7. Any perceived benefits of the lock? (If yes, what?)
8. Any challenges that may be encountered? (If yes, what?)
9. Would you use the system, would you upload your face for improved access?
10. Do you think this device would be of use at your place of work?
11. How would this work for use in domiciliary care? Care in the community?

**Thank participants, email them the below text as a debrief (copy and paste).**

Thank you for taking the time to complete this interview, we appreciate your input.

To confirm, the purpose of this study was to inform the evaluation of the Touchbyte facial recognition lock service.

Please be assured your answers will only be viewed by the research team, and any reports will use summarised data and not your individual answers. Your interview audio will be stored securely. You will not be named or identifiable in any work produced and all of your data, including email address, will not be shared any third parties including for fundraising purposes.

Please feel free to contact us with any questions via email at epic@plymouth.co.uk. Thank you again for your participation.

**Post-implementation interview schedule**

Interview schedule prompts:

1.       How was the Touchbyte lock introduced to you?

**Probe:**Why do you feel it was introduced at this site?

**Alternative**: How did you implement the Touchbyte lock at your site?

2.       How has your experience been of using the lock?

**Probe:**Did the lock match your expectations?

3.       What, if any, benefits did you experience as a result of using the lock?

**Probe:** were there any benefits to customers or the site more broadly, e.g. has the lock altered the customer experience, security, safety, workflow, speed of access? Were there any unintended consequences of using the lock? *2

4.       Were there any barriers or concerns for you using the lock? If so, what were they and could they be overcome in anyway?

**Probe:**safety, ethics, privacy, time to learn, lack of awareness, non-engagement, no appropriate technologies, lack of support or training, trust, firewalls, wifi, anxiety)

**5.**Has the lock changed the way you work in anyway? If so, how?

**Probe:** Were you able to use the lock as much as you wanted to? Is so/not, why? How does the Touchbyte lock compare with the standard entry system?

**6.**Did you feel supported in using the lock?

**Probe:**Did you receive training for using the lock? How was your training experience? Could the implementation or introduction of the Touchbyte lock be improved in anyway?

7.       Could the lock or system itself be improved in anyway?

**Probe**: could it be made more accessible? More user-friendly? Content? Safety? Quality? Design?

8.      Is there anything that you would recommend to other sites or healthcare professionals looking implement or use the Toucbyte lock? If so what, and why?

9. Would you consider continuing to use the Touchbyte lock? If yes/no, why?

10. Is there anything specific, beyond what we have covered, that you think needs further exploration? (e.g. time saving, carbon emissions, less travelling)

11. What comments / feedback has there been from regular or one-time visitors to the site regarding Facentry?

Probe: Although they may not have used the system, have these visitors noticed and asked about it? Have they expressed any views? For example, have relatives that regularly visit their loved ones expressed any views on their perceptions of how the system might change things for their loved ones and for them? (e.g. perception of safety, security and convenience)

12. If the establishments could use Facentry on access points in addition to the front door, what would those be and why?

13. When using the camera to access, how long has it generally taken? Do they feel they have had to wait? If so, what length of wait seems reasonable before frustration creeps in?

14. Regarding the light: Have users seen it? What was it doing? What did they think it meant? Did it tell them anything? If so what? Did they use it to understand if the door had opened? What did they think it was for? Had anyone explained what it did? What ideas do they have to make it better or replace it / add to it?

15. Finally, any other comments, thoughts or experiences we haven’t covered?

**A2: Prompts for process mapping**

Process Mapping Prompts

- Role:
- Process under discussion: (e.g. entering care home before/after face recognition).
- Define the start point. What input (triggers) starts the process? (e.g. need to access)
- Define the end-point (e.g. entered building)
- What are the outputs or process steps? (estimate times for each step and between each step)
- Who are the receivers of the outputs?
- Identify all staff groups involved in each stage
- What are the outcomes of this process?
- Repeat for process for before and following implementation of technology.

**Table A3: Displaying content analysis and example evidence from pre-implementation interviews**

**Table A4: Displaying content analysis and example evidence from post-implementation interviews**

**Table A3: Displaying content analysis and example evidence from pre-implementation interviews**

The themes and codes are provided with a frequency of occurrence in brackets. Quotes are also provided with participant identifiers.

| **Theme** | **Grouped codes** | **Initial codes** | **Example Evidence** |
| --- | --- | --- | --- |
| Concerns with current system (92) | Current system – security concerns and inefficiency for community care (16) | Key box issues (4), Control undesirable access (5), Ease of carer/NHS access considering mobility or hearing impairments and code sharing issues (7), | “Because keys getting lost or the key codes being shared between multiple workers causes a risk to that person within the community” (P1)  “We do a lot of driving. So of course, when we get there, we want to be able to get in, just want to get, get on with what you're there for.” (P6) |
|  | Current system -security concerns for care homes (12) | Security issues with codes (4), Safety of residents (6), Poor GDPR (2) | “Some of our residents are quite smart, they watch you. And then they're going to play with the codes. And sometimes they work them out by fluke.” (P1)  “With codes as well, in some services, they'll have the same code. So ones that I know, they've had the code, they've been the same on all of the external doors for at least three years.” (P2) |
|  | Current system – safety and security concerns for care homes (14) | Covid spread (10), Code known by many users (3), Broken systems (1) | “So if you've got if you got a PIN code on your on your door, and nobody cleans after every use, then actually it's just a point of entry. And so when we had the second wave of COVID, last November, we didn't know what hit us. […] so we lost quite a lot of people again” (P3) |
|  | Current system – Inefficiency concerns for care homes (50) | Delay in entering (11), Staff don’t know codes (6), Having to learn codes (6), Doorbell or card failures (3), Hands full (2), Institutional (1), Time costly relaying codes to agency (2), Rust (1), Time lag (2), NHS visitors inefficient access (2), Visitors stuck inside (4), Staff too busy to attend door (4), lost key fobs (1), Onerous (1), Inaccurate visitor log (3), Visitor feels burdensome (1) | “So quite often, you're left standing there for quite a long time. And then you're kind of left wondering whether anyone's heard you, or whether the doorbells even working. Quite often we'd stand there and think, I wonder if that doorbell is working. And then you obviously try knocking but you don't, you don't want to be too intrusive. Like, you don't want to feel like you're kind of harassing whoever's inside, if they're, if they're busy, you know, doing important things caring for people, you don't want to be kind of harassing them by banging on the door or ringing the doorbell, you know, 15 times,” (P8) |
| Benefits of current system (14) | Current system –benefits for care homes and community (14) | Low cost (2), No Covid risk (3), Already keys in place (1), Autonomy for community care (2), No delays (1), Everyone knows current system (3), Control access (2) | “I guess the pin-pads, they don't cost anything to maintain, do they once they're there, they're there. So it's kind of more of a one off cost” (P3) |
| Benefits to FRLT (36) | FRLT benefit –safety for care homes and community care (5) | Benefit – thinking about spread of Covid (4),  More secure care (1) | “[Lock could be] increasing the safety of our homes, both for our staff and residents, safety with regards to entry and exit of our buildings, access to restricted areas and the safer method and the IPC [infection control] reduction.” (P1) |
|  | FRLT benefit – Efficiency for care homes and community care (27) | Staff not knowing codes due to code changes, flexi staff, agency, visiting staff (8),  Family and friends ease of visit (2), Accurate staff logging (3), Saving staff time (7), Doctors ease of visit (1), Saves visitor waiting time (4), Reduce lost cards (1), Hands free access (1) | “Well, but then if, say if people arrive so if the caterers arrival, the laundry arrive, and they don't know the pin number. And then you've got someone who's going to let people in and having to stop what they do. And right now, that means they've got to wash their hands, sanitize themselves, open the door, let somebody in make sure they do the same. Whereas the facial recognition detection system. It would warrant there being the additional cost, because you're saving time for staff that are having to answer the door and having to come away from residents when they could be providing care. And so the savings are going to make the cost worth it.” (P3) |
|  | FRLT benefit – safety and efficiency in community care (4) | Emergency (1), Ease of access (3), End of life (1) | “Sometimes we need to see people every day of the year, 365 days of the year as well. So that would be great, just to be able to go get diabetics, people are often on a six month injection to prevent blood clots, things like that. So for people where it's kind of longer term, then it could be it could be a value [when visiting] those patients.” (P6) |
| FRLT suggestions (10) | FRLT potential additions or improvements (10) | Temperature camera (1), Integrate with other systems (7), Let visitors out also (1), Notify internal staff (1) | “At the moment we sort of, we come to the building and we punch in the code, then we've got to take our phones out, start up the [staff clock-in] app, log into [the app], which can be an issue with the Wi Fi here sometimes. And then we log in. And that's how we get paid. So by coming up to the door with the camera, then opening the door and logging us on to the system would save us quite valuable time actually” (P4) |
| Concerns with FRLT (90) | FRLT concerns – technological (25) | Changes to facial features (4), Depends on electricity (cost) (3), Power cuts (2), Internet (5), Device failures (3), Weather (5), Flashing light - epilepsy (1), Staff without smartphones (1), Night time (1) | “You can't necessarily use that in the rain, or if it's dark. So I guess all of those constraints would also be there for for their camera.” (P5)  “And if you have a power cut, nothing works unless it's almost like a backup. So that would be that, that's the only thing that's sort of jumping out is saying there was a power cut” (P2) |
|  | FRLT concern – logistics for care homes (26) | High volume of users (9), Admin required (4), System wide approach (1), Many cameras required (2), Staff learning the system (1), Price (6), People accessing without staff control (3), | “One challenge I get, I can foresee is that we would need to establish a framework with our agency providers that they ensure that they get they gave us the required facial selfies, so that we could upload the facial recognition of the agency worker to the camera, because that could be one of the barriers that we that I foresee. And what we would need to do is make sure that there is a quick way of updating it. Especially the high turnover. agency staff. So for me, one barrier or problem that I foresee is that it needs to be reactive enough to be able to work by changing for workforce. Yeah.” (P1) |
|  | FRLT concern – logistics for community care (24) | Community logistics, many visitors (8), Price (11), Emergency (1), poor staff retention (4) | “There would be a number. Yeah, be a number of people. And as things open up more services go in. Then obviously there's lots of outlying occasional people like podiatrists, like speech and language, they might only see someone once yeah But they need to go and assess the swallow I say on somebody who had a stroke, and things like that. ” (P6) |
|  | FRLT concerns – perceptions and data security (12) | Attitudes towards the technology (7) Consent from those who use it (1), Privacy, attitudes, spying (2), Unsettling (2) | “I'd be looking for some kind of assurance that my face wasn't going to be used for any other purposes.” (P8) |
|  | FRLT concern – safety (3) | Emergency (3) | “So I think Who else would I think oh, and then this is obviously if we get an emergency like an ambulance or, or something like that. […]the ambulance drivers and paramedics in Cornwall get a special card that they wave in front of the camera and lets them in” (P3) |
| FRLT Acceptability | Current FRLT/biometrics acceptability (4) | FRLT on phone (2), Previous experience biometrics (2), | “So I have no issue with sort of biometric data, my phone uses facial recognition to unlock.” (P2) |
|  | Personal acceptability to proposed FRLT | Would use system at work?   Would upload own face? | “I think it's great. It's great. I mean, it's great not to have to touch something, and it would be a lot easier for someone like myself that could just go brilliant. I could just go to there.” (P7) |

**Table A4: Displaying content analysis and example evidence from post-implementation interviews**

| **Theme** | **Grouped codes** | **Initial codes** | **Example Evidence** |
| --- | --- | --- | --- |
| Benefits of FRLT for residential care settings (53) | Improved customer or resident autonomy (7) | Accessible without staff, autonomy (6), well adopted by residents (1) | “I just think it's nice, it's a nicer experience for them. Our customers, isn't it where, you know, I think sometimes they don't necessarily need to have staff presence to go and open a door. And they can just go straight up to where they need to go. And it gives them more independence.” (P12) |
|  | Improved efficiency for care settings (23) | Saving staff time (8), avoiding delays waiting at door and faster access for staff and customers (6), financial savings (2), too many codes to remember (3), hands free (2), cost (1), responsive to staffing changes (1) | “Because sometimes I mean, we do have a gentleman at the moment, two to one. So when he's in the vicinity has to be two staff, because you cannot just go and answer the door and leave him with one person say, yeah, there probably is a delay, you know, and it is frustrating cuz we're all watching the clock.” (P12)  “I was gonna say it’s cost effective, and with time management is cost effective. It frees up more time for us to be doing the workload that we have, rather than like I said earlier, I often answer the door, sometimes, like you answer, then you answer. And it's always a constant flow. So with the face recognition in place, it just gives us a lot more freedom to, to continue working.” (P13) |
|  | Improved security (10) | No code sharing, access only for those approved (5), no code guessing, safer for residents (2), quicker access in dangerous situations (1), staff turnover (1), different access to different areas for different team members (1) | “Some of our customers may be prone to be picked on. And even if someone is following them, we know [with the FRLT], that person can get in the door. Which is nice. Because no matter how much we try and protect, outside, there's always going to be something okay, but it's nice to have once they're in the door. Nobody can get in that door, if they've also been following them. I think that's one of the nicest things for me. So I think it's a factor of safety for them. Because there will always be someone that wants to pick on somebody. And it's just the fact they get in even if they're being followed, they get in the door quickly” (P14)  “Security with staff that change their careers. So as staff leave, it makes the system more responsive.” (P9) |
|  | Improved safety (13) | Accurate log of who entered building (4), better GDPR than manual log (1), less infection control touchpoints (8) | “But then it become more accurate. Yeah. Exactly. Who's in the building?” (P6)  “I think the other benefit that I could really see is from an infection control point of view, because it's unlocking the door with the, you know, without having to touch a key or touch a, you know, like a key code pad. So that's gonna be a significant benefit.” (P8) |
| Positive reactions (27) | Reliable (7) | Reliability (3), works with mask on (1), manageable system, intuitive light system (4), | “Yeah, no. I mean, I'm I'm in here virtually seven days a week. So I used to live next door until Saturday. So even on my days off, I used to pop in for a cup of coffee. So yes. So since it's been there, I think I've been in here virtually every day. Yeah. And I think if I remember right, is only once that it went red and then I waved, and it went green straight away.” (P13)  “That was really good in the dark as well, cuz obviously it goes green in the dark. So when it's night time, I feel like, yeah, it's still visual for the guys and the staff. Yeah, I was coming in like midnight. So it's pitch black. They've [customer] been to the pub and had one too many. It's still that green visual. I mean, I'm safe. And it still works in the dark” (P12)  “It does work with my face mask up, which is a bonus.” (P4) |
|  | Widespread potential (13) | More widespread potential (7), use in domiciliary care (3), use for more independence (1), use for different doors to allow access to different people (2) | “I certainly think for the future of this type of supported living schemes and, and similar. I like the idea that again, it again, it gives people a bit more autonomy, it gives a security of a building. So you know, nobody can just walk in only people that are allowed in can get in.” (P13)  “I've seen [community] carers unlock a key safe and leave the key safe open, they take the key with them into the house, but anybody walking by that, you know, you just go go look at the co-ordinates, and then you know, what it is to unlock. So, you know, if, say, I was an opportunist thief, I could then sort of go and get the code. And then at any point that I want to enter that building, I've got access to the key. Whereas something like the [FRLT] system, you know, we could load the photos up or any family members, any professionals that might sort of be visiting so like community nurses, district nurses, things like that”(P8)  “it's got a massive potential to to make life a lot easier around the inside of the home, not just the actually the exit of the home.” (P4)  “I think I think for me, it's it's more if this was rolled out, I think I think that would be really good. Yeah. Yeah, I would say it should be more widely used” (P1) |
|  | Adoption (7) | Would recommend (1), well adopted (2), would continue using (4), high usage, positive family opinion (1) | “I would like to see it being quite widely used in in care homes, and in domiciliary care as well. I think there'd be quite a strong application for there in people's homes, where maybe they do have like a lockbox or something like that. It could definitely improve security in those situations. Yeah, it's just it's just not you know, in COVID times, it's just nice not to have to touch anything.” (P2)  “I would say, all the staff that work here, are all aware of it, they're all using it.” (P2)  “I've got no negativity. I mean, so demo was positive for us” (P10)  “Our gentlemen have got a hold of it brilliantly” (P11)  “Customers love it.” (P13) |
| Barriers encountered (43) | Perceived concerns (11) | Power cuts (1), robustness (4), trust in GDPR (2), no infection control benefit (1), no guidance on where to go once in (1), logistics of uploading staff long-term (1), cost (1) | “We had a couple of issues I don't know if you're aware a little old lady come down. Off the Wall. Yeah, she come down and like ripped off the wall. Yeah. Yeah. She doesn't live here. No, no, no, no, no, she got dementia.” (P12)  “Yeah, yeah, that's the thing, isn't it? It's the cost. Because not only is it the whole system, but it's got to be fitted. And yes, yeah, there's probably quite big costs that.” (P1)  “Because people are different height. You know, I know [team member] initially he had to find a bracket to put on it because the sun was glaring. But yeah, I think that's probably why it's broken a few times because people have tried to adjust it to fit their position.” (P13) |
|  | Negative reactions (13) | Did not match expectations (1), frustrations (2), not quick enough (5), slower than touchpad (5), | “I can see there being a benefit. It just needs I think tweaking slightly to make it more user friendly and quicker. That's that's the main issue, really, I mean, because the girls, they sort of come in, they're on a mission, they want to get in, get started, get changed, get all scrubbed up and stuff. So they'll go up to the facial recognition camera, then they've got a wave, and then they're trying to get their face and it won't, it will flash red.” (P1)  “I mean, with all new technology, there's always going to be sort of hiccups and glitches and things like that. There are a few things I've noticed about the system. It's like, sometimes you have to wave your hand in front of it, to get it to activate and then it reads your face and let's you in. And I have noticed a lot of the carers are too impatient. They prefer to just quickly tap the keypad because it's quicker than using the face recognition system.” (P4) |
|  | Problems encountered (19) | Camera not at right height (2), sun glare issue (4), issues in positioning face, customer lack of understanding, requirement for training, positioning advice needed (4), didn’t work (6), hand waving (1), Removing mask (2) | “Two of our gentlemen have got a hold of it brilliantly. But we have got one that we support that's struggling with that. And I think what he's trying to do is he's putting his face right up to it. And I think is getting too close. I think we need to go out there and teach him how to use it properly.” (P14)  “Think if there's a bit of glare from the sun, maybe that might affect it.” (P4)  “We've sent over numerous numerous photographs, but nothing ever works. Right? So he's out there doing this and it's never, ever worked.” (P13) |
| Suggested improvements (18) | Accessibility considerations (8) | Audio feedback (2), positioning feedback (1), screen to display your positioning (1), camera moves to find your face (1), not understanding lights (1), Hearing impairment and colour blindness (2) | “Maybe some audio feedback. So like I said, obviously, this is probably going to work for hearing unable to people, but yes, and kind of, I don't know. Yeah, audio feedback, like, move, even if it could say like, move forward, or step back, or unlocked, or something like that. I think that would be quite helpful” (P8)  “So if it had a screen where you can see your face on it, because for me, that looks like it's too low. Yeah. It doesn't, yes, if you could see that it was it was seeing on like a feedback screen. And like, you know, I don't know, some of the face facial recognition things you can, you've got to put it within a frame, like on the screen. So at least you could line yourself up with it. Straight away” (P9) |
|  | Integration with other systems (10) | Visitor log-book (1), staff clock-on system (3), fire system (2), healthcare visitors, exit camera (1), vaccination status (1), car reg for emergencies (1), Track and Trace (1) | “We also were saying about the fire register, because obviously, getting people signed in is quite difficult because the families don't. Okay. So if we had to face recognition, where it would recognize family members, then that would be good for us for fire registers as well.” (P10)  “And what I would like you to be able to do is with the changes around the government guidelines, is when we set up a professional or a staff account that we as as a care provider and as for example, as a manager of that home site, utilize your system as our due diligence around COVID vaccination status, say that only those with active use have confirmed vaccination status.” (P9)  “Eventually, in the future, it could link to [staff clock-on app] that would get the carers massively on side, because at the moment, they come in, they punch in the keypad, they open the door, they get their mobile phone now they log in to the app and they, punch in well if you said to the carers right. Okay, all you've got to do now is walk up to the camera, it will let you in and it punches you into the clock-on system that would get a lot of them on side a lot quicker.” (P2)  “Track and Trace. That is actually. That would be really good.” (P1) |
